# RNA-Seq Transcriptome Analysis Provides Candidate Genes for Resistance to *Tomato Leaf Curl New Delhi Virus* in Melon

**DOI:** 10.3389/fpls.2021.798858

**Published:** 2022-01-18

**Authors:** Cristina Sáez, Alejandro Flores-León, Javier Montero-Pau, Alicia Sifres, Narinder P. S. Dhillon, Carmelo López, Belén Picó

**Affiliations:** ^1^Institute for the Conservation and Breeding of Agricultural Biodiversity, Universitat Politècnica de València, Valencia, Spain; ^2^Cavanilles Institute of Biodiversity and Evolutionary Biology, Universitat de València, Valencia, Spain; ^3^World Vegetable Center, East and Southeast Asia, Research and Training Station, Kasetsart University, Nakhon Pathom, Thailand

**Keywords:** melon (*Cucumis melo* L.), ToLCNDV resistance, transcriptome (RNA-seq), DEG (differentially expressed genes), qPCR (quantitative polymerase chain reaction)

## Abstract

Tomato leaf curl New Delhi virus (ToLCNDV) emerged in the Mediterranean Basin in 2012 as the first DNA bipartite begomovirus (*Geminiviridae* family), causing severe yield and economic losses in cucurbit crops. A major resistance *locus* was identified in the wild melon accession WM-7 (*Cucumis melo* kachri group), but the mechanisms involved in the resistant response remained unknown. In this work, we used RNA-sequencing to identify disease-associated genes that are differentially expressed in the course of ToLCNDV infection and could contribute to resistance. Transcriptomes of the resistant WM-7 genotype and the susceptible cultivar Piñonet Piel de Sapo (PS) (*C. melo* ibericus group) in ToLCNDV and mock inoculated plants were compared at four time points during infection (0, 3, 6, and 12 days post inoculation). Different gene expression patterns were observed over time in the resistant and susceptible genotypes in comparison to their respective controls. Differentially expressed genes (DEGs) in ToLCNDV-infected plants were classified using gene ontology (GO) terms, and genes of the categories transcription, DNA replication, and helicase activity were downregulated in WM-7 but upregulated in PS, suggesting that reduced activity of these functions reduces ToLCNDV replication and intercellular spread and thereby contributes to resistance. DEGs involved in the jasmonic acid signaling pathway, photosynthesis, RNA silencing, transmembrane, and sugar transporters entail adverse consequences for systemic infection in the resistant genotype, and lead to susceptibility in PS. The expression levels of selected candidate genes were validated by qRT-PCR to corroborate their differential expression upon ToLCNDV infection in resistant and susceptible melon. Furthermore, single nucleotide polymorphism (SNPs) with an effect on structural functionality of DEGs linked to the main QTLs for ToLCNDV resistance have been identified. The obtained results pinpoint cellular functions and candidate genes that are differentially expressed in a resistant and susceptible melon line in response to ToLCNDV, an information of great relevance for breeding ToLCNDV-resistant melon cultivars.

## Introduction

Melon (*Cucumis melo* L.) is one of the major cucurbit crops cultivated worldwide and with large diversification. It is highly appreciated for its nutritional profile and its sweet and aromatic flavor, and its production generates large profits to farmers in developing and industrialized countries.

Despite years of selection and breeding, melon production around the globe is compromised by several pathogens and diseases. The occurrence of new viruses emerging in melon-growing regions is a major concern for farmers and seed producers because of the yield-limiting potential of these biological agents (Annu et al., 2019).

*Tomato Leaf curl New Delhi virus* (ToLCNDV) is a species of bipartite begomovirus (family *Geminiviridae*) naturally transmitted by the whitefly *Bemisia tabaci* (Gennadius) in a persistent manner. This virus has posed a major threat to melon crops in many countries of the Indian subcontinent since the earliest 2000s, but recently, a new recombinant strain has appeared in the Mediterranean basin that has generated a devastating disease to mainly melon and zucchini squash (*Cucurbita pepo*) crops (Juárez et al., 2014). The new strain was first identified in Spain, but all the isolates detected across the Mediterranean countries share a conserved genomic sequence (Juárez et al., 2014, 2019; Fortes et al., 2016; Panno et al., 2019). Consequently, the Mediterranean ToLCNDV strain has been designated as ToLCNDV-ES (Moriones et al., 2017).

Similar to the Asian strains, ToLCNDV-ES infection in melon results in severe symptomatology, including curling and distortion of leaves, green and yellow spotting conforming mosaic, and changes in leaf shape and stunting. Infection at young stages of the plant hampers growth and flowering with decreased or complete loss of fruit quality due to skin roughness, longitudinal cracking, and small fruit size, rendering them unmarketable (Panno et al., 2016). Melon production losses may reach 80% in greenhouse and open-field conditions if integrated control measures against the disease are not adopted (Messelink et al., 2020). For ToLCNDV-ES management, genetic resistance is the most efficient approach for farmers; it reduces the need for chemical treatments and, thus, is safer for producers, consumers, and the environment.

Melon accessions belonging to the Indian momordica, kachri, acidulous, and agrestis groups are reported to be resistant to begomoviruses (Yousif et al., 2007; McCreight et al., 2008; López et al., 2015; Pitrat, 2016; Romay et al., 2019; Martín-Hernández and Picó, 2021), and several QTLs associated with resistance have been identified in this germplasm (Sáez et al., 2017; Romay et al., 2019).

In previous works, we mapped a major gene with incomplete dominance conferring resistance to ToLCNDV-ES on chromosome 11 of the Indian wild melon accession WM-7 (kachri group, landrace originated from the semiarid region of Punjab state, India) (Roy et al., 2012; Sáez et al., 2017). However, two minor modifiers on chromosomes 2 and 12 modulate the response to this viral infection. Functional characterization of the genes located in these regions is required to enhance understanding of the molecular resistance mechanisms in WM-7 to ToLCNDV.

Transcriptome sequencing using RNA-seq technology has gained popularity to explore gene expression changes in cucurbit plants during viral infections (Li et al., 2017a; Sun et al., 2017, 2019; Lou et al., 2020). This tool offers a global view of expression changed during the basal defense response and helps to elucidate complex resistance mechanisms in plants through comparing gene expression upon infection in susceptible and resistant lines.

Transcriptome analysis and gene expression studies have been performed to evaluate interactions between ToLCNDV in solanaceous crops. Sahu et al. (2010) compare transcript levels between tomato genotypes tolerant and susceptible to ToLCNDV, finding induced genes in the tolerant genotype related to the cell cycle, transcription factors (TFs), DNA/RNA processing, and molecular signal and transport. The overexpression of two of the main candidate genes for resistance, a DEAD-box RNA helicase gene (*SlDEAD35*) and the 26S proteasome subunit RPT4a (*SlRPT4*), involved in the hypersensitive response and cell death, respectively, is associated with the inhibition of ToLCNDV infection and symptom expression (Sahu et al., 2016; Pandey et al., 2019). In pepper (*Capsicum annuum*) and potato (*Solanum tuberosum*) NBS-LRR genes are found induced in resistant genotypes after ToLCNDV infection (Kushwaha et al., 2015; Jeevalatha et al., 2017).

Román et al. (2019) measured expression differences of 12 candidate genes between a ToLCNDV resistant and a susceptible melon accession. Two genes, an a NAC TF and an actin related protein, were strongly induced in the susceptible genotype and were associated with disease development. Apart from those studies, global transcriptional and molecular characterization of ToLCNDV resistance in cucurbits has not been conducted.

In this study, we performed an RNA-seq assay to compare transcript levels between mock-inoculated and ToLCNDV-ES-infected melon plants between the resistant WM-7 genotype and a susceptible accession from day 0 to 12 days after treatment to identify candidate genes for resistance.

## Materials and Methods

### Plant Material and Mechanical Inoculation

The ToLCNDV-resistant wild accession WM-7 (*C. melo*, kachri group) and the susceptible traditional Spanish cultivar Piñonet Piel de Sapo (PS) (*C. melo*, ibericus group) were used as plant materials in this study. WM-7 is an Indian accession (Roy et al., 2012), selfed in successive growing cycles to increase the level of homozygosity. Seeds of both accessions were disinfected in a 10% sodium hypochlorite solution for 1 min and washed twice with distilled water for 5 min. To ensure homogenous germination, all seeds were opened using forceps, placed over moistened cotton in Petri dishes and incubated in the dark at 37°C for 48 h. Seedlings were transplanted into pots containing commercial substrate (Humin-substrat N3, Neuhaus Klasmann-Deilmann, Germany) and growth in a climate chamber at 25°C and 60% relative humidity under a photoperiod of 16 h/8 h (day/night).

At the two true-leaves stage, 108 seedlings of both resistant and susceptible accessions were mechanically inoculated with ToLCNDV-ES following the procedure described in López et al. (2015). Symptomatic leaf tissue from zucchini plants agroinfiltrated with an infective ToLCNDV-ES clone (Sáez et al., 2016) was used as the inoculum source. The tissue was mashed in an iced mortar together with inoculation buffer in a 1:4 (*w:v*) proportion (López et al., 2015), and the mix was scrubbed with a cotton swab over one cotyledon and one true leaf, previously dusted with Carborundum 600 mesh. The same number of plants were mock-inoculated following the same protocol but rubbing with only inoculation buffer and Carborundum. Seedlings were cultivated for 20 days after mechanical inoculation (dpi) under controlled conditions as described above.

### Sampling Design

At 0 dpi (two true-leaves stage), all expanded leaves of six healthy seedlings were sampled and used as the control treatment, maintaining seedlings alive with their apex intact. The remaining seedlings were mock- or virus-inoculated and sampled following the same procedure as the 0 dpi control but avoiding the inoculated leaves. Six different seedlings were sampled at each stage corresponding to 3, 6, 9, 12, 15, and 18 dpi. Samples were frozen immediately in liquid nitrogen before storage at –80°C. Once sampled, all plants were maintained until 20 dpi to test virus accumulation or absence by tissue-printing hybridization as described below. Then, the tissue of the six plants of each time point was pooled to a single sample. For each time point, three biological replicates were processed independently.

After evaluation of viral load by quantitative polymerase chain reaction (qPCR), samples of two biological replicates at 0, 3, 6, and 12 dpi were selected to be included in the RNA-seq assay, corresponding to a total of 28 samples (Table 1). The third replicate was included in qRT-PCR validation along with the two sequenced biological replicates.

**TABLE 1:** Summary of the RNA-Seq experimental design, read alignment, and differentially expressed genes (DEGs).

Sample name	Inoculation treatment	Time point dpi	Replicate	Cleaned reads	Mapped reads	% Mapped reads	DEGs
WM-7_0dpi_1	Uninoculated	0	1	96,571,093	92,933,140	96.23	–
WM-7_0dpi_2		0	2	89,738,162	86,520,235	96.41	
WM-7_I3dpi_1	ToLCNDV inoculated	3	1	96,017,670	92,139,780	95.96	292
WM-7_I3dpi_2		3	2	90,412,987	87,094,110	96.33	
WM-7_B3dpi_1	Mock inoculated	3	1	97,337,221	93,893,779	96.46	
WM-7_B3dpi_2		3	2	90,083,950	53,101,818	58.95	
WM-7_I6dpi_1	ToLCNDV inoculated	6	1	95,502,062	88,222,091	92.38	354
WM-7_I6dpi_2		6	2	89,625,183	74,603,092	83.24	
WM-7_B6dpi_1	Mock inoculated	6	1	96,044,324	92,625,513	96.44	
WM-7_B6dpi_2		6	2	90,375,930	86,463,397	95.67	
WM-7_I12dpi_1	ToLCNDV inoculated	12	1	96,637,024	92,925,369	96.16	558
WM-7_I12dpi_2		12	2	90,324,017	86,939,559	96.25	
WM-7_B12dpi_1	Mock inoculated	12	1	97,444,807	94,078,242	96.55	
WM-7_B12dpi_2		12	2	91,063,342	87,570,888	96.16	
PS_0dpi_1	Uninoculated	0	1	102,027,470	95,339,552	93.44	–
PS_0dpi_2		0	2	90,578,076	87,236,559	96.31	
PS_I3dpi_1	ToLCNDV inoculated	3	1	95,503,001	91,778,653	96.1	404
PS_I3dpi_2		3	2	60,411,004	57,587,132	95.33	
PS_B3dpi_1	Mock inoculated	3	1	97,063,008	93,062,108	95.88	
PS_B3dpi_2		3	2	92,073,644	88,433,510	96.05	
PS_I6dpi_1	ToLCNDV inoculated	6	1	96,678,397	92,088,929	95.25	752
PS_I6dpi_2		6	2	93,655,611	89,233,728	95.28	
PS_B6dpi_1	Mock inoculated	6	1	96,653,614	92,903,742	96.12	
PS_B6dpi_2		6	2	91,746,137	88,232,317	96.17	
PS_I12dpi_1	ToLCNDV inoculated	12	1	95,935,797	92,131,302	96.03	981
PS_I12dpi_2		12	2	91,460,724	87,827,401	96.03	
PS_B12dpi_1	Mock inoculated	12	1	96,368,610	92,693,418	96.19	
PS_B12dpi_2		12	2	89,485,860	85,736,511	95.81	

*Assigned name to each sample, their temporal and inoculation treatment, and replicates performed are shown. Proportion of uniquely clean reads mapped against the reference melon genome (v4.0), and differentially expressed genes comparing firstly with 0 dpi control and then between mock and ToLCNDV inoculated treatments are provided.*

### Evaluation of Response to *Tomato Leaf Curl New Delhi Virus*

Symptoms of ToLCNDV were assessed in all plants at each sampling stage and at 20 dpi, according to the visual scale described in López et al. (2015), in which score 0 means absence of symptoms and score 4 very severe symptoms. Quantitative PCR (qPCR) was used to determine the viral titer evolution at the different stages analyzed (0, 3, 6, 9, 12, 15, and 18 dpi). Total DNA was extracted from each pooled sample of inoculated WM-7 and PS accessions in the three biological replicates using the Cetyltrimethyl ammonium bromide (CTAB) method (Doyle and Doyle, 1990). ToLCNDV amount was analyzed by qPCR in two technical replicates, following the same procedure described in Sáez et al. (2017).

Additionally, to verify the presence or absence of ToLCNDV infection in all plants at 20 dpi, tissue-printing hybridization by digoxigenin-labeled ToLCNDV-RNA probe was performed following the procedure described in Sáez et al. (2021).

### RNA Extraction

The 28 samples of processed leaf tissue were sent to the Genomics4All Company (Universidad Politécnica de Madrid, Spain) for cDNA library construction. Total RNA extraction and DNAse treatment was performed with TURBO DNA-free™ kit (Ambion, Life technologies), following the manufacturer’s protocol. RNA was purified with RNeasy Kit columns (Qiagen), and RNA integrity was checked on a Bioanalyzer 2100 (Agilent Technologies, Santa Clara, CA, United States) to ensure an RNA integrity number (RIN) score of ≥7. Total RNA (500 ng) were used to prepare RNA-seq libraries using the TruSeq Stranded mRNA Library Prep Kit (Illumina) according to the manufacturer’s protocol.

For qRT-PCR validation, total RNA was isolated using 700 μL of Extrazol^®^ EM30 (Blirt DNA, Gdansk, Poland), according to the manufacturer’s specifications. Integrity was checked by 1% agarose gel electrophoresis, and purity and quantity were determined spectrophotometrically at 260 and 280 nm using a NanoDrop 1000 (Thermo Scientific, Waltham, MA, United States). Total RNA (1 μg) treated with PerfeCTa^®^ DNase I (RNAse-free) (Quanta Biosciences, Gaithersburg, MD, United States) was used as template with the RevertAid™ First Strand cDNA Synthesis Kit (ThermoFisher Scientific) with Oligo dT primers.

### RNA-Seq Data Analysis and Evaluation of Expression Differences

RNA-seq libraries were sequenced (single-end 50 pb) using a HiSeq 2500 Illumina (Illumina, CA, United States). Raw sequences were processed using Trimmomatic (Bolger et al., 2014) to remove adapters and low-quality reads. Quality of clean reads was checked by FastQC (Andrews, 2010).

Trimmed reads were mapped to the last version of the melon reference genome (v.4.0), recently updated and publicly available at the melonomics.net website (Castanera et al., 2020), using STAR v. 2.02.01 (Dobin et al., 2013). Subsequently, RSEM v. 1.3.1 was used to quantify the abundance of transcript reads assigned to each of the 28.299 genes included in the complete genome annotation according genome assembly v. 4.0 (Li and Dewey, 2011).

Clusters of genes sharing similar expression profiles across all samples were obtained by performing a weighted gene co-expression network analysis (WGCNA), using the R package WGCNA v.1.69 (Langfelder and Horvath, 2008). To test for statistical differences due to the effects of genotype, treatment, and time after inoculation, a generalized linear model using the cluster’s eigengenes was performed. Kyoto Encyclopedia of Genes and Genomes (KEGG) enrichment analysis (Kanehisa and Goto, 2000) of genes that were significantly correlated at *p-value* < 0.01 with each gene cluster was performed using *clusterProfiler* (Yu et al., 2012).

Differentially expressed genes (DEGs) were detected using the DESeq2 package (version 1.26.0) (Love et al., 2014), comparing raw counts of samples at 3, 6, or 12 dpi against 0 dpi. Genes with *p-values* under 0.05 and | log2 (fold changes)| ≥ 1 were considered as significant, assigning those presenting log2 (fold change) ≥ 1 to the “upregulated” group and those with values ≤ –1 to the “downregulated” group, compared to the 0 dpi stage.

Regarding the background “noise” encountered in previous time course transcriptomic experiments (Nueda et al., 2012) and with the view that eliminating as many false positives from the final data set is key (Conesa et al., 2016; Giacomini et al., 2018), we performed an additional filtering step to optimize and add ruggedness to our analysis. In this approach, the expression DEGs data sets obtained after comparing expression levels of 3, 6, and 12 dpi time points to 0 dpi were then further compared between mock and ToLCNDV inoculated samples at the same dpi stage, selecting those DEGs differing by at least ± 1.5 log2 (fold change) values between ToLCNDV and mock treatments. Thus, developmental changes of the melon plant along time are discarded, and DEGs obtained here must be attributed to ToLCNDV infection in each genotype.

Common DEGs at the different stages of the disease and those shared between genotypes were represented by Venn diagrams, displayed with the jvenn tool (Bardou et al., 2014), freely available at http://bioinfo.genotoul.fr/jvenn.

The Cucurbits Genomics Database (CuGenDB^1^) was employed to determine biological functions and pathways of the identified DEGs. GO term enrichment analysis was performed uploading DEGs lists, considering significantly enriched those with an adjusted *p-value* < 0.05.

### Validation of Differentially Expressed Genes by Quantitative Polymerase Chain Reaction

To validate the RNA-Seq data, expression of seven candidate genes putatively associated to ToLCNDV resistance in melon was evaluated by qRT-PCR in six out of the seven time points sampled (0, 3, 6, 9, 12, and 18 dpi). Confirmation of the expression patterns of these seven genes was performed in the three biological replicates of mock and viral inoculated plants. cDNA for the validation experiment was prepared as described in section “RNA extraction.”

PCR reactions were performed on a Roche LightCycler^®^ 480 RT-PCR System (Roche Diagnostics, Rotkreuz, Switzerland) using the FastStart Essential DNA Green Master (Roche Molecular Systems, Rotkreuz, Switzerland). Each qPCR reaction contained 1.5 μL of cDNA, 7.5 μL of FastStart Essential DNA Green Master (Roche Diagnostics, Rotkreuz, Switzerland) and 1.5 μL (10 μM) of each primer and of H_2_O to a final volume of 15 μL. Primer design was made using Primer 3Plus software (Untergasser et al., 2007), and primer sequences are listed in Supplementary Table 1. The corresponding specific fragment was amplified in two technical replicates following these conditions: 95°C for 5 min, 40 cycles of 95°C for 15 s, 60°C for 30 s, and 72°C for 15 s. A melting curve analysis (60–95°C) was performed after the amplification to check specificity of the reaction. Expression levels were analyzed using relative quantitative accumulation by the 2^(−ΔΔCt)^ method (Schmittgen and Livak, 2008). As endogenous controls, the melon Peptidyl-prolyl *cis-trans* isomerase gene (Cyclophilin CYP7, MELO3C025848.2) (González-Ibeas et al., 2007), was used as a reference for the expression level of each candidate gene in every condition.

### Transcript-Based Single Nucleotide Polymorphism Identification

To study the potential effect of genetic changes in coding regions, reads were aligned against the reference *C. melo* genome using *bowtie2* version 2.3.4.1 (Langmead and Salzberg, 2012), and variant-calling of Single Nucleotide Polymorphism (SNPs) and Indels was performed by Freebayes version 1.3.4 (Garrison and Marth, 2012), setting a minimum mapping quality cutoff of 40, minimum base quality of 20 and minimum minor allele frequency of 0.05. Variant annotation and its predicted functional effect on the transcript were made using SNPEff version 5.0e (Cingolani et al., 2012).

## Results

### Assessment of WM-7 and PS Response to *Tomato Leaf Curl New Delhi Virus* Infection

**FIGURE 1:**
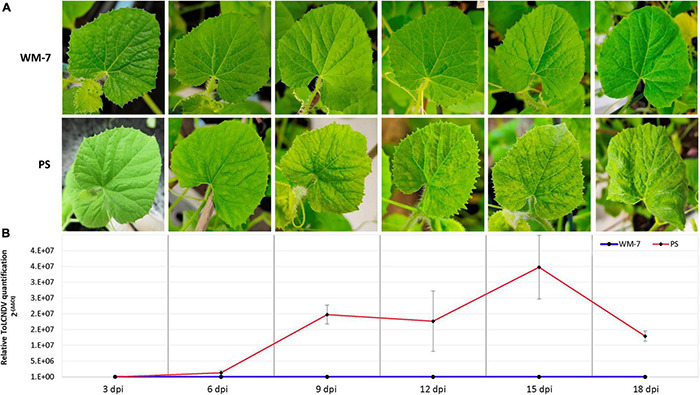
Assessment of WM-7 and PS response to ToLCNDV-ES infection in the first total expanded apical leaf of each plant. Temporal evolution of symptomatology **(A)** and mean of viral titers **(B)** in WM-7 and PS.

All WM-7 plants inoculated with ToLCNDV remained symptomless throughout the assay (Figure 1A). Conversely, early mild symptoms (score 2) were identified in PS at 6 dpi on a small number of plants. This score increased at 9 dpi. At 12, 15, and 18 dpi all PS plants showed severe symptoms (scores from 3 to 4) including mosaic, curling, and yellowing (Figure 1A).

To quantify the relative viral accumulation of ToLCNDV in both WM-7 and PS after infection, a qPCR assay was performed, using healthy plants (0 dpi) as controls. ToLCNDV was identified at all stages in both genotypes except at 0 dpi. Viral accumulation was low in both WM-7 and PS at the early stage of 3 dpi, but significant differences were observed in the course of the disease (Figure 1B). Whereas WM-7 viral accumulation levels remained low until the end of the assay, viral load in PS continued to increase at 6 and 9 dpi, achieving the highest accumulation at 15 dpi (Figure 1B).

Qualitative viral titers at 20 dpi were determined by tissue-printing hybridization (data not shown). No signal was detected in control and mock-inoculated WM-7 and PS plants, whereas in all PS infected plants, there was a high level of ToLCNDV. Only some WM-7 plants exhibited slight hybridization signals, suggesting low viral titers.

According to these results, ToLCNDV was capable of initially infecting both resistant and susceptible genotypes. However, virus replication and/or movement were restricted in WM-7, whereas in PS, the infection became systemic after 6 dpi.

### RNA Sequencing, Reads Alignment, and Weighted Gene Co-expression Network Analysis

Twenty-eight libraries were sequenced, generating almost 2.6 billion high-quality clean reads. On average, 94% of the clean reads were mapped to the melon reference genome (v4.0) unambiguously to a single locus (Table 1). Only in one sample, corresponding to the second replicate of ToLCNDV inoculated WM-7 at 6 dpi (WM7_I6dpi_Rep2), 58.95% of reads mapped to a single locus as a consequence of the high content of ribosomal RNA in this sample. This effect was corrected by the STAR outfilter multimap nmax function, selecting only those reads mapping to a single locus (Table 1). The 28 transcriptomes were further analyzed to compare gene expression of resistant and susceptible genotypes.

**FIGURE 2:**
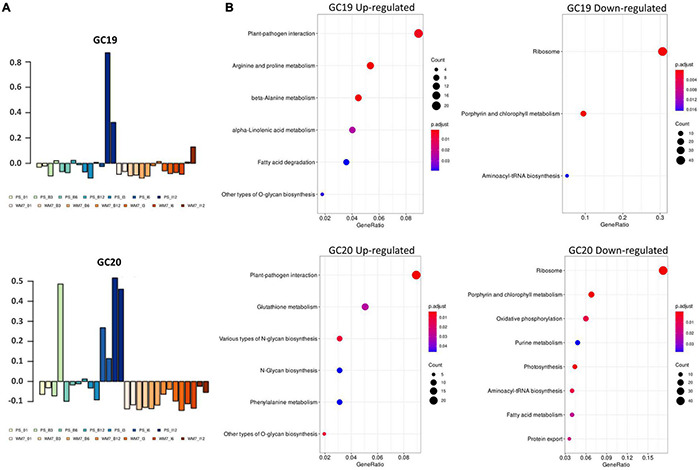
Module eigengenes across samples for the gene clusters GC19 and GC20 **(A)**. KEGG enrichment profile of up- and down-represented genes within each cluster **(B)**. I: ToLCNDV inoculated; B: mock inoculated; 01: 0 dpi, 3: 3 dpi, 6: 6 dpi, 12: 12 dpi.

Weighted gene co-expression network analysis produced 21gene clusters (GCs) based on the expression profiles of the 28 samples (Supplementary Figure 1). Genes associated to each GC as well as KEGG and GO term enrichments are presented in Supplementary Tables 2, 3, respectively. Some of the clusters clearly show a genotype-specific pattern of gene co-expression (e.g., GC 8, 16, and 21) or plant age (e.g., GC4 and 7). But only GCs 19 and 20 showed statistically significant differences regarding genotype × treatment × time (Figure 2A and Supplementary Table 4). Most upregulated genes within these GCs were enriched in KEGG pathways associated to plant–pathogen interaction, whereas those with an expression negatively correlated with the clusters were mainly classified in “Ribosome” and “Photosynthesis” ontologies (Figure 2B). Further, GO ontologies including upregulated co-expressed transcripts in GC19 and GC20 were “Transmembrane transport” in biological process and “ADP binding” and “Calmodulin binding” in molecular function (Supplementary Table 3). Conversely, “Hydrolase activity” and “GTP binding” of the molecular function class and “Chloroplast” and “Thylakoid” in the category cellular components included genes downregulated (Supplementary Table 3).

### Differential Expression Analyses and Functional Classification by Gene Ontology Enrichment

**FIGURE 3:**
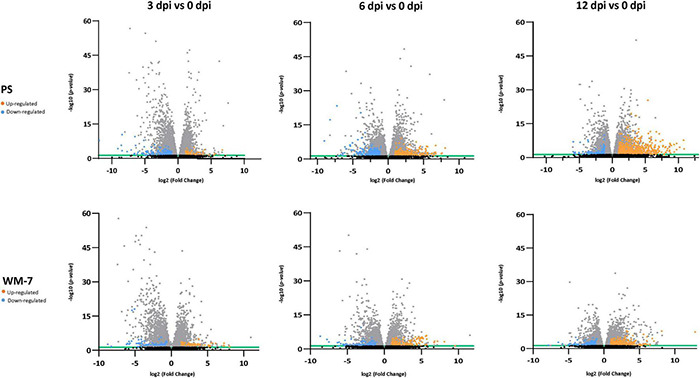
Volcano plots display DEG distribution for each time point at 3, 6, and 12 dpi in both PS and WM-7. Orange dots represent the upregulated genes and blue dots downregulated genes. The *x*-axis correspond to log2 (fold change) and the *y*-axis to –log10 (*p*-adjusted). Black dots represent those DEGs not considered significant with *p*-adjusted ≥ 0.05 (green line shows the cutoff) and gray dots those DEGs differing from mock-inoculated treatments in 1.5 < log2 (fold change) > 1.5.

In total, 1204 genes were differentially regulated in the resistant WM-7 genotype and 2137 genes in the susceptible PS genotype. The number of DEGs increased with the course of the disease for both genotypes (Table 1, gene lists are in Supplementary Tables 5, 6). At 3 dpi, the lowest number of DEGs was observed for both WM-7 and PS. At this stage, all plants were asymptomatic and with low viral titers regardless the genotype. Interestingly, at all stages the number of DEGs was higher in PS than in WM-7, and both genotypes showed a different pattern of gene expression (Figure 3). Whereas the number of upregulated genes in PS plants increased markedly as infection progressed (159, 312, and 800 upregulated genes at 3, 6, and 12 dpi, respectively), in WM-7, this increase was less profound (146, 222, and 343 DEGs, respectively). Contrastingly, the number of downregulated genes in PS did not show this increasing pattern with a maximum at 6 dpi (245, 440, and 181 downregulated genes at 3, 6, and 12 dpi, respectively) although a slight increase was found in WM-7 (146, 132, and 215 DEGs). These results suggest that ToLCNDV infection causes a lower impact on the transcriptomic reorganization in WM-7 than in PS, consistent with similar studies comparing the response to begomoviruses in resistant and susceptible accessions (Allie et al., 2014; Zaidi et al., 2020).

**FIGURE 4:**
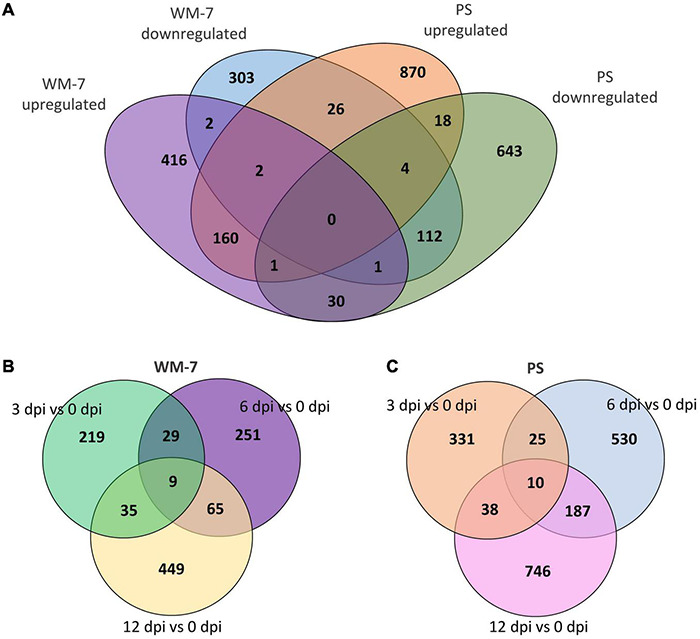
Venn diagrams representing common and specific up and down DEGs identified in WM-7 and PS **(A)**; common and specific DEGs at 3, 6, and 12 dpi in WM-7 **(B)** and PS **(C)**.

PS and WM-7 shared 336 DEGs during ToLCNDV infection (Figure 4A). Interestingly, 56 of these DEGs had an opposite expression pattern with 26 induced in the susceptible genotype and repressed in the resistant and 30 upregulated in WM-7 but downregulated in PS (Figure 4A and Supplementary Table 7). Both sets of common DEGs include genes related to resistance to pathogens, host–pathogen systems, and components required to accomplish viral infection.

**FIGURE 5:**
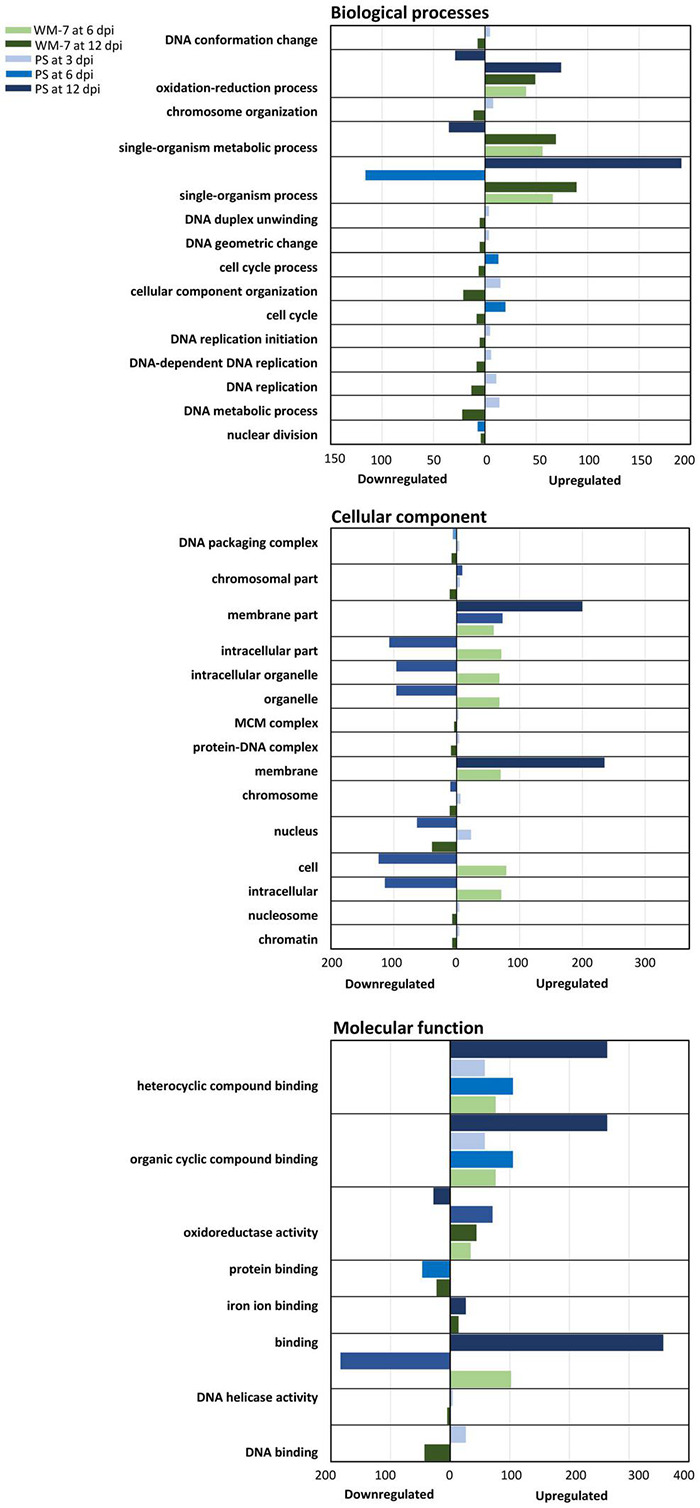
Gene ontology (GO) classification. The number of upregulated or downregulated genes assigned to each class were calculated at 3, 6, and 12 dpi to WM-7 and PS and represented in colors according with the legend.

To further analyze and understand this response, an analysis of gene ontologies was performed, identifying GO terms of DEGs shared between the resistant and susceptible genotypes (Figure 5). Among the biological process group, those terms involved in chromosome organization and replication, metabolism, and conformation of DNA, contained DEGs that were induced at 3 dpi in PS but were repressed in WM-7 at 12 dpi. In the cellular component category, among the upregulated genes, those encoding membrane components were the most enriched in PS at 6 and 12 dpi. Although there were WM-7 membrane-associated genes overexpressed too, the number of these genes in PS was much greater than in the resistant genotype. A high number of upregulated genes in WM-7 concerned intracellular organelles; a group of these was repressed in PS at 6 dpi. Genes codifying proteins located in the nucleus or chromosome organization were induced in PS at 3 dpi but down-expressed at 6 dpi. By contrast, transcription of these genes in WM-7 was suppressed at 12 dpi. Although less in number represented, genes of the minichromosome maintenance protein complex (MCM) were repressed in WM-7 at 12 dpi and upregulated in PS at 3 dpi. MCM proteins interact with Rep protein of geminiviruses to facilitate their replication (Rizvi et al., 2015). Examining the GO terms concerning molecular components, those related with “binding” were the most enriched in PS, showing genes with highly altered expression patterns. Some genes related to DNA helicase activity were induced early in PS (3 dpi) but repressed at 12 dpi in WM-7.

Gene ontology terms including DEGs at 3 dpi in WM-7 and also in PS at any time point were not identified. REVIGO (Supek et al., 2011) was used to group similar GO term categories exclusively identified in WM-7 or PS but not in both lines. Scatterplots were constructed to present these results in Supplementary Figure 2. In total, 721 genes were exclusively found expressed in WM-7 and 1531 genes in PS (Figure 4A). In WM-7, nine of these genes showed differential expression at the three time points (Figure 4B and Supplementary Table 8), seven were upregulated, and two downregulated. A multiprotein-bridging factor 1c (MELO3C004553.2) on chromosome 5 showed the highest expression differences at 3, 6, and 12 dpi [log2 (fold change) of 3.525, 3.419, and 4.071, respectively]. A similar response was observed for a gene codifying a cysteine proteinase inhibitor (MELO3C002921.2) on chromosome 9. The cysteine proteinase inhibitor gene family has been described conferring resistance to viruses in plants (Gutiérrez-Campos et al., 1999; Gholizadeh et al., 2005). Two cysteine-rich receptor-kinase-like protein genes (CRKs) (MELO3C018796.2 on chromosome 1 and MELO3C002492.2 on chromosome 12) presented both strongly induced and repressed expression patterns at different stages in infected WM-7 plants. CRK involvement in pathogen resistance and cell death in plants has been well described (Lu et al., 2017; Yadeta et al., 2017; Quezada et al., 2019). Additionally, another nine genes related to heat stress responses were altered at some of the three stages, and a great number of photosynthetic genes had enhanced expression at 6 and 12 dpi (Supplementary Table 5). Another gene (MELO3C027682.2, chromosome 7) encoding a DNA-directed RNA polymerase, which has been associated with defense responses (Nemchinov et al., 2016), was also remarkably overexpressed at 6 and 12 dpi (Supplementary Table 8).

In PS plants, 10 genes were found to be DEGs at the three evaluated stages of ToLCNDV disease development (Figure 4B and Supplementary Table 9). The expression profiles of two of them, an accelerated cell death 6-like protein (MELO3C004399.2, chromosome 5) and a cysteine-rich receptor-like kinase (MELO3C018799.2, chromosome 1), were especially interesting as they were highly downregulated at 3 dpi but changed to be overexpressed at 6 and 12 dpi when this genotype developed symptoms and systemic infection of the virus, conversely to what was found in WM-7 for some of these types of genes.

### Analysis of Differentially Expressed Genes in the Candidate Regions for Tomato Leaf Curl New Delhi Virus-ES Resistance

We focused our expression analyses on those genes included in the three candidate regions of the *C. melo* genome linked to the resistance to ToLCNDV-ES, at the major resistance QTL on chromosome 11, and on the minor QTLs on chromosome 2 and 12 (Sáez et al., 2017).

#### Transcription Changes on Chromosome 11

The main locus involved in the resistance identified in WM-7 was located in chromosome 11 between positions 30,112,560 and 30,737,924 bp. In this interval, seven genes were found to be differentially expressed in PS and three in WM-7 (Table 2).

**TABLE 2:** Differentially expressed genes in the candidate region for ToLCNDV resistance of *C. melo* chromosome 11.

			Log2 (Fold Change)				
	Gen	Stage dpi	ToLCNDV inoculated	Mock inoculated	Start	End	Function
**PS**	MELO3C022339.2	6	–3.08	–	30,494,799	30,495,239	Glutaredoxin
	MELO3C022300.2	12	2.179	–	30,156,612	30,160,071	Probably inactive receptor-like protein kinase At2g46850
	MELO3C022324.2	12	1.888	–	30,394,274	30,397,789	Proton pump-interactor 2-like
	MELO3C022341.2	12	10.806	–	30,509,871	30,512,205	Bidirectional sugar transporter SWEET
	MELO3C022358.2	12	1.451	–	30,691,737	30,692,270	Ethylene-responsive transcription factor ERF113-like
	MELO3C022365.2	12	1.743	–	30,715,606	30,716,293	UPF0481 protein
	MELO3C022367.2	12	1.168	–	30,725,006	30,743,460	UPF0481 protein At3g47200
**WM-7**	MELO3C022327.2	3	–2.809	–	30,409,446	30,410,934	Transmembrane protein, putative
	MELO3C022337.2	3	–1.075	–	30,481,662	30,482,590	Auxin-responsive protein SAUR36
	MELO3C022327.2	6	–3.596	–	30,409,446	30,410,934	Transmembrane protein, putative
	MELO3C022319.2	12	–1.075	–	30,347,186	30,355,934	DNA primase large subunit

Only one out of the seven genes in PS, a gene encoding a Glutaredoxin protein (MELO3C022339.2), was downregulated at 6 dpi; the other six genes were upregulated, especially at 12 dpi. The highest induced expression was detected for a bidirectional sugar transporter SWEET (MELO3C022341.2) with a log2 (fold change) of 10.806. Genes with a lower induction were a proton pump-interactor 2-like isoform X2 (MELO3C022324.2), described as a plasma membrane regulator (Bonza et al., 2009), and an ethylene-responsive TF ERF113-like (MELO3C022358.2) protein family reported as induced in infected plants with TYLCV (Wu et al., 2019). Between the other three induced genes, MELO3C022365.2 and MELO3C022367 encode uncharacterized proteins, and MELO3C022300.2 is a probably inactive receptor-like protein kinase.

In WM-7, three DEGs identified in the candidate region for resistance of chromosome 11 were downregulated (Table 2). A transmembrane protein, putative (MELO3C022327.2) was repressed at 3 and 6 dpi, whereas Auxin-responsive protein SAUR36 (MELO3C022337.2) was repressed only at the beginning of ToLCNDV-ES infection (3 dpi). Auxins and jasmonic acid (JA) pathways are interconnected, and both regulate defense response to begomoviruses, particularly to ToLCNDV in tomato (Ramesh et al., 2017; Vinutha et al., 2020). Further interesting was a downregulated at 12 dpi DNA primase large subunit (MELO3C022319.2), protein family associated with geminivirus replication (Gutierrez, 2000; Guilliam et al., 2015). DEGs identified in this region must be regarded as the main candidate genes implicated in triggering ToLCNDV infection and in the defense response in melon.

#### Transcription Changes on Chromosomes 2 and 12

Candidate regions for ToLCNDV-ES resistance in chromosomes 2 and 12 were larger than the region of the major QTL (Sáez et al., 2017), and hence, a larger list of DEGs was obtained for both regions (Supplementary Table 10).

In chromosome 2, many of the DEGs were TFs and disease-related proteins downregulated in PS at 3 dpi, but subsequently upregulated at 6 and 12 dpi. In the case of NAC (MELO3C017185.2) and bHLH35 (MELO3C017424.2) TFs, high induction was observed in PS after 6 dpi. A transmembrane protein (MELO3C017283.2) was highly induced at 12 dpi in PS, whereas at 3 dpi, a phosphoethanolamine *N*-methyltransferase (MELO3C017356.2) implicated in DNA methylation was upregulated. In WM-7, photosynthetic proteins were highly induced at 6 and 12 dpi.

A second QTL linked to ToLCNDV-ES resistance was mapped at the top of chromosome 12 (Sáez et al., 2017; Supplementary Table 10). In this region, a cytochrome P450 gene (MELO3C004742.2) was strongly repressed at 3 dpi in PS, whereas the gene Photosystem II protein D1 (MELO3C027714.2) located in the same region was over-expressed in WM-7 at 12 dpi.

Mitogen-activated protein kinases are proteins implicated in the signal pathway of JA biosynthesis and interact with MYC2. Overexpression of these proteins induces salicylic acid (SA) and JA gene expression and promotes TYLCV resistance (Li et al., 2017b). MELO3C026848.2 encodes a mitogen-activated protein kinase, which was downregulated in the susceptible genotype PS at the beginning of infection (3 dpi). Also located in this candidate region, a NAC-type TF (MELO3C004694.2) was highly induced in PS at 12 dpi, while a DNA-directed RNA polymerase II subunit E (MELO3C021758.2) was downregulated in WM-7 at 6 dpi.

### Expression of Known Genes Related to Defense Response

To further investigate and identify candidate genes contributing to ToLCNDV resistance or susceptibility, we analyzed the expression profile of 70 R-genes of melon previously characterized by Islam et al. (2020) and other categories of genes related to the defense response in geminivirus infection, including gene silencing, pathogen-resistant proteins, and hormonal response.

#### R-genes

Of the characterized 70 R-genes in *C. melo*, 19 of them, distributed on chromosomes 1, 3, 4, 5, 7, 9, and 12 were differentially expressed in the susceptible genotype, whereas only three of them, located on chromosomes 4, 5, and 11, presented an altered expression in the resistant genotype (Table 3). In PS, all DEGs followed the same trend with repressed expression at 3 dpi but upregulation at 6 and 12 dpi. Similarly, in WM-7, one R-gene with LRR domain on chromosome 11 was strongly repressed at 6 dpi near the candidate region of resistance to ToLCNDV-ES described in Sáez et al. (2017), but the remaining two (MELO3C009694.2 and MELO3C004309.2) were upregulated at 12 dpi.

**TABLE 3:** Differentially expressed genes identified between the 70 R-genes of the melon genome characterized in Islam et al. (2020).

		log2 (Fold Change)						Gene				
		3 dpi		6 dpi		12 dpi						
		ToLCNDV inoculated	Mock inoculated	ToLCNDV inoculated	Mock inoculated	ToLCNDV inoculated	Mock inoculated	Chromosome	Start	End	Function	Functional disease resistance-related domains
**PS**	MELO3C023579.2			1.305	–	1.492	–	1	32,573,956	32,576,619	Disease resistance protein RGA2-like isoform X1	LRR
	MELO3C023578.2	–1.063	–					1	32,588,913	32,593,637	Disease resistance protein	NB-ARC
	MELO3C023441.2	–1.232	–					1	33,623,112	33,627,816	Receptor-kinase, putative	LRR
	MELO3C010826.2					3.548	–	3	28,214,790	28,218,285	Receptor-kinase, putative	LRR
	MELO3C010825.2					6.754	4.610	3	28,218,855	28,226,256	Receptor-kinase, putative	LRR
	MELO3C009693.2			1.112	–	1.407	–	4	28,945,912	28,948,344	Disease resistance protein	NB-ARC
	MELO3C009179.2			1.429	–			4	32,531,221	32,534,345	Receptor-kinase, putative	LRR
	MELO3C004289.2	–1.145	–			1.062	–	5	25,863,643	25,869,731	TMV resistance protein N-like	TIR-NBS-LRR
	MELO3C004303.2			1.697	–	1.664	–	5	26,036,017	26,040,632	TMV resistance protein N-like	LRR
	MELO3C004311.2			1.560	–	2.700	–	5	26,076,967	26,095,083	TMV resistance protein N-like	TIR-NBS-LRR
	MELO3C004313.2			2.177	–	1.771	–	5	26,106,563	26,109,785	TMV resistance protein N-like	TIR-NBS-LRR
	MELO3C017703.2	–1.062	–					7	24,126,814	24,129,634	Disease resistance protein RGA2-like	NB-ARC
	MELO3C022146.2					2.410	–	9	744,523	750,028	TMV resistance protein N-like	TIR-NBS-LRR
	MELO3C022144.2			1.048	–	1.406	–	9	767,040	775,410	TMV resistance protein N-like	TIR-NBS-LRR
	MELO3C005450.2			2.717	–	3.153	–	9	20,427,143	20,430,013	LRR receptor-like kinase family protein	LRR
	MELO3C005451.2			5.330	–	7.293	3.030	9	20,434,381	20,437,380	LRR receptor-like kinase	LRR
	MELO3C002506.2			1.722	–	3.179	–	12	22,229,817	22,238,994	Receptor-like protein kinase	RLK
	MELO3C002504.2	–1.634	–			2.234	–	12	22,242,891	22,252,225	Cysteine-rich receptor-like protein kinase 28	RLK
	MELO3C002501.2					2.578	–	12	22,262,412	22,265,400	Cysteine-rich receptor-like protein kinase 26isoform X1	RLK
**WM-7**	MELO3C009694.2					1.795	–	4	28,938,791	28,941,261	Disease resistance protein	NB-ARC
	MELO3C004309.2					2.092	–	5	26,060,336	26,066,739	TMV resistance protein N-like	TIR-NBS-LRR
	MELO3C022449.2			–4.838	–			11	31,293,359	31,296,020	Receptor-like protein	LRR

*Expression pattern of each gene is presented as log2 (fold change) at 3, 6, and 12 dpi stages. Physical position of genes according to v4.0 of melon genome, functional annotation, and disease resistance–related domain are included.*

*TIR-NBS-LRR: Toll/interleukin-1 receptor homology nucleotide-binding site leucine-rich repeat. Function: pathogen specificity and defense.*

*LRR: Leucine-rich repeat. Function: Recognition of pathogen and Plant Defense.*

*NB-ARC: Nucleotide-binding adaptor shared by APAF-1, R proteins and CED-4. Function: molecular switch in activating defenses.*

*RLK: Protein kinase. Function: Signaling and plant.defense.*

The expression pattern of other genes annotated with a resistance to pathogens function was also studied (Table 4). Most of these genes presented an altered expression pattern in PS as shown in the R-genes described above, whereas WM-7 exhibited a more diverse regulation pattern. In both genotypes, pathogenesis-related protein 1 (PR-1) family genes were altered. These proteins are the most abundantly produced when pathogens infect plants and have been considered as hallmarks of hypersensitive response and broad-spectrum systemic acquired resistance in association with salicylic acid (SA) (Balint-Kurti, 2019; Guerrero et al., 2020).

**TABLE 4:** Expression pattern of additional selected genes conferring resistance to pathogens.

		log2 (Fold Change)						Gene				
		3 dpi		6 dpi		12 dpi			Chr	Start	End	Function
		ToLCNDV inoculated	Mock inoculated	ToLCNDV inoculated	Mock inoculated	ToLCNDV inoculated	Mock inoculated					
**PS**	MELO3C018539.2					6.232	–		1	1,023,454	1,024,330	Pathogenesis-related protein 1-like
	MELO3C018540.2					4.757	–		1	1,030,810	1,031,348	pathogenesis-related protein 1-like
	MELO3C018544.2			1.148	–				1	1,042,545	1,043,212	Pathogenesis-related protein 1
	MELO3C018547.2					2.416	–		1	1,051,651	1,052,250	Pathogenesis-related protein 1
	MELO3C018878.2					3.820	–		1	3,559,447	3,560,191	Pathogen-induced protein CuPi1
	MELO3C023694.2					3.590	–		1	6,394,591	6,396,233	Pathogen-related protein
	MELO3C023578.2	–1.063	–						1	32,588,913	32,593,637	Disease resistance protein
	MELO3C017322.2	–1.803	–						2	24,275,353	24,280,275	LEAF RUST 10 DISEASE-RESISTANCE LOCUS RECEPTOR-LIKE PROTEIN KINASE-like 1.2 isoform X2
	MELO3C008149.2			1.310	–	2.130	–		3	1,163,259	1,172,229	LEAF RUST 10 DISEASE-RESISTANCE LOCUS RECEPTOR-LIKE PROTEIN KINASE-like 1.4 isoform X1
	MELO3C031443.2					1.049	–		5	18,385,406	18,392,554	TMV resistance protein N
	MELO3C004262.2			4.967	–	7.110	–		5	25,614,803	25,621,533	TMV resistance protein N-like
	MELO3C004265.2					–3.999	–		5	25,650,358	25,651,288	TMV resistance protein N-like isoform X2
	MELO3C004291.2			3.994	–	5.272	–		5	25,906,332	25,908,625	TMV resistance protein N-like
	MELO3C031332.2	6.714	4.701						5	26,375,103	26,376,034	Disease resistance protein
	MELO3C004354.2			1.032	–	1.137	–		5	26,592,987	26,596,766	CC-NBS-LRR resistance protein
	MELO3C004385.2					5.488	–		5	26,848,627	26,849,611	Pathogenesis-related protein PR-4-like
	MELO3C019482.2			–1.096	–				6	10,571,172	10,573,959	Pathogenesis-related thaumatin-like protein
	MELO3C016941.2					1.139	–		7	947,005	948,144	Protein ENHANCED DISEASE RESISTANCE 2
	MELO3C017692.2					4.161	–		7	24,081,397	24,084,555	Disease resistance protein RGA2-like
	MELO3C017703.2	–1.062	–						7	24,126,814	24,129,634	Disease resistance protein RGA2-like
	MELO3C033615.2			1.350	-	1.808	–		9	6,658,398	6,659,268	Disease resistance protein RGA2-like
	MELO3C029858.2	–1.042	–						9	20,748,481	20,749,427	NBS resistance-like protein
	MELO3C020880.2			–1.323	–				11	3,294,068	3,294,968	Pathogenesis-related protein 1
	MELO3C024731.2					3.364	–		11	7,170,632	7,173,642	Disease resistance protein RGA2-like
	MELO3C002084.2			2.412	–	2.657	–		12	24,991,237	24,991,695	Protein NEGATIVE REGULATOR OF RESISTANCE
**WM-7**	MELO3C018796.2	–2.636	–			–6.437	–		1	2,864,020	2,864,927	Cysteine-rich receptor-kinase-like protein
	MELO3C018540.2	5.297	–			5.506	–		1	1,030,810	1,031,348	Pathogenesis-related protein 1-like
	MELO3C018539.2					5.704	–		1	1,023,454	1,024,330	Pathogenesis-related protein 1-like
	MELO3C012701.2			1.438	–				1	21,798,030	21,798,830	Cysteine proteinase inhibitor
	MELO3C012702.2			1.869	–				1	21,826,195	21,827,118	Cysteine proteinase inhibitor
	MELO3C017497.2					5.171	–		2	22,319,516	22,320,537	Pathogenesis-related protein PR-1
	MELO3C008149.2					1.128	–		3	1,163,259	1,172,229	LEAF RUST 10 DISEASE-RESISTANCE LOCUS RECEPTOR-LIKE PROTEIN KINASE-like 1.4 isoform X1
	MELO3C031111.2	1.548	–	1.533	–				5	7,065,995	7,068,688	Negative regulator of systemic acquired resistance SNI1
	MELO3C004261.2					2.559	–		5	25,594,168	25,594,730	TMV resistance protein N-like
	MELO3C004309.2			2.056	–	2.092	–		5	26,060,336	26,066,739	LOW QUALITY PROTEIN: TMV resistance protein N-like
	MELO3C016128.2			–1.373	–				7	19,211,072	19,212,632	Pathogen-related protein
	MELO3C017071.2			1.514	–				7	123,638	125,187	Cysteine/Histidine-rich C1 domain family protein
	MELO3C022150.2					2.260	–		9	703,234	708,384	TMV resistance protein N-like
	MELO3C033944.2	–3.577	–7.025			–4.180	–		9	16,122,393	16,123,197	NBS-LRR type resistance protein
	MELO3C002921.2	1.250	–	1.610	–	1.477	–		9	7,641,728	7,642,167	Cysteine proteinase inhibitor
	MELO3C002492.2	4.266	–	3.409	–				12	22,299,334	22,300,624	Cysteine-rich receptor-like protein kinase 25

Some defense-related proteins were upregulated at 6 and 12 dpi in PS, including seven tobacco mosaic virus (TMV) resistance genes (chromosomes 5 and 9) and several TFs, such as NAC, MYB, bHLH, genes of hormonal response to ethylene, and a negative regulator of resistance protein (Supplementary Table 9).

#### Transcription Factors

In geminivirus infections, TFs involved in plant development are differentially regulated (Kumar, 2019). We searched for DEGs coding TFs, and 17 MYB were altered at all stages in both genotypes (Supplementary Tables 5, 6). In PS, 23 DEGs were of WRKY type, and 16 were NAC TFs, all differentially regulated at mainly 6 and 12 dpi. Out of 16 NACs with altered expression, 12 were upregulated (Supplementary Table 5). Conversely, only four WRKY genes showed transcription changes in WM-7, one repressed at 3 dpi (Supplementary Table 6). In this genotype, there were also three altered NAC TFs; out of them, two were downregulated (Supplementary Table 6). Only one MYC-2 TF (MELO3C022250.2) was suppressed at 6 dpi in chromosome 11 of PS (Supplementary Table 5). MYC-2 belongs to the basic helix-loop-helix (bHLH) TF family and interacts with the BV1 protein of bipartite begomoviruses (Li et al., 2014). This protein directly regulates terpene transcription genes and mediates whitefly resistance achieving vector-virus mutualism. A member of the terpene cyclase/mutase genes family (MELO3C022374.2) was strongly suppressed in PS at 6 dpi but highly induced in WM-7 at 12 dpi (Supplementary Table 7). Similarly, 11 DEGs were bHLH in PS at 6 or 12 dpi (seven downregulated). In WM-7 plants, six bHLH genes were identified, all upregulated when compared with healthy controls. Therefore, ToLCNDV-ES generates a stronger readjustment of TF expression in PS than in WM-7.

#### Hormones

Lipoxygenases (LOX) are essential enzymes required in the JA hormone synthesis (Wasternack and Song, 2017) and associated with systemic defense, hypersensitive response, and cell death when pathogens infect plants (Hwang and Hwang, 2010; Vicente et al., 2012). LOX genes differentially regulated have been reported after geminivirus infection (Góngora-Castillo et al., 2012; Allie and Rey, 2013). In the susceptible and resistant genotypes, 13 and 6 LOXs presented altered expression at all stages, respectively. Interestingly, all the genes coding these proteins were located in two clusters on chromosome 5 (Supplementary Tables 5, 6). Among them, four were common DEGs to PS and WM-7 (MELO3C004244.2, MELO3C014630.2, MELO3C027325.2, and MELO3C031318.2) (Supplementary Table 7) and were induced early in the resistant genotype but suppressed in the susceptible melon.

Salicylic acid and JA pathways promote resistance to begomoviruses and also to heat stress, and both responses are mediated by common gene families (Tsai et al., 2019). The geminivirus coat protein interacts with heat shock proteins (HSPs) and recruits them to their own viral regulation, interfering with the host antiviral response (Gorovits et al., 2013; Gorovits and Czosnek, 2017; Jeevalatha et al., 2017; Kumar, 2019). HSP downregulation in resistant plants restricts begomovirus movement through plasmodesmata (Naqvi et al., 2017). However, out of 19 and 32 HSPs differentially regulated in PS and WM-7, 17 and 30 had a strong induction, respectively (Supplementary Tables 5, 6).

Some defense-related proteins were upregulated at 6 and 12 dpi in PS, including genes of hormonal response to ethylene and a negative regulator of resistance protein (Supplementary Table 9).

#### Ubiquitination and Ubiquitin/Proteasome System Complex

In plants, ubiquitination contributes to resistance to geminivirus (Czosnek et al., 2013). RING-type E3 ubiquitin ligase and F-box genes are components of the proteasomal ubiquitination complex (UP) and have been described interacting with begomovirus infection (Correa et al., 2013). Nine RING-type E3 ubiquitin ligase genes were altered in PS, five of them downregulated and four induced, whereas only one RING-type E3 ubiquitin transferase (MELO3C003458.2) was repressed in WM-7 (Supplementary Tables 5, 6). F-box genes were altered in both susceptible and resistant genotypes. In PS, eight F-box genes were induced and five underexpressed, and similarly, three F-box genes had repressed expression in WM-7 and four were induced (Supplementary Tables 5, 6). F-box proteins regulate diverse cellular processes, including cell cycle transition, transcriptional regulation, and signal transduction, and mediate ubiquitination of proteins targeted for degradation by the proteasome, playing an essential role in many cellular processes.

#### RNA Silencing

As RNA silencing constitutes one of the major strategies to develop resistance against geminiviruses, we searched for DEGs involved in this mechanism.

Calmodulin proteins regulate the RNA-silencing machinery, and their induction increases geminivirus accumulation in plants (Chung et al., 2014). Calmodulin proteins can reduce the expression of RNA-dependent RNA polymerase (RDRs) proteins and interact with suppressor of gene silencing 3 (SGS3) and degrade it by autophagy. Despite the fact that Calmodulin binding was one of the enhanced GO categories in GC19 (see section “RNA sequencing, reads alignment and WGCNA”), we detected only four calmodulin DEGs downregulated at 3 dpi in PS and four upregulated at 6 and 12 dpi. In WM-7, just one calmodulin gene was induced at 12 dpi (Supplementary Tables 5, 6). One autophagy protein (MELO3C031521.2) was also upregulated in WM-7 at 12 dpi.

Expression of two cytosine-specific methyltransferases (CMT3), implicated in transcriptional gene silencing (TGS), was altered in PS and WM-7 (Supplementary Tables 5, 6). In the susceptible genotype, the gene MELO3C015649.2 on chromosome 2 encodes this kind of enzyme and was repressed at 6 dpi. Conversely, a gene with similar function (MELO3C026448.2, chromosome 10) was induced early at 3 dpi in WM-7. CMT3 also interacts with autophagy and ubiquitin pathways (You et al., 2019). Two cytosine-specific methyltransferases are also deregulated: one induced in WM-7 at 3 dpi (MELO3C026448.2) and one repressed in PS at 6 dpi (MELO3C015649.2) (Supplementary Tables 5, 6). These enzymes have homology with MET1, also a methyltransferase involved in TGS.

Chromatin and histone methylation are mechanisms used by plants to regulate gene expression of invasive viral DNAs (Castillo-González et al., 2015). A histone-lysine *N*-methyltransferase (MELO3C025676.2) was induced in PS at 3 dpi, whereas the same gene was repressed in WM-7 at 12 dpi (Supplementary Table 7). In the resistant WM-7 genotype, two additional Histone-lysine *N*-methyltransferase genes, MELO3C011304.2 and MELO3C012115.2, were, respectively, induced and repressed at 12 dpi (Supplementary Table 6). A histone acetyltransferase coding gene (MELO3C011266.2) was also repressed at 3 dpi in this genotype. Additionally, in the course of assay, seven genes codifying histones appeared downregulated, and two were induced in the resistant accession. In PS (Supplementary Table 5), MELO3C022387.2 another histone-lysine *N*-methyltransferase was induced at 3 dpi in chromosome 11. A histone acetyltransferase and a histone demethylase (MELO3C018028.2 and MELO3C017723.2, respectively) were also repressed in this genotype at 6 dpi, and 10 genes coding histones were deregulated, five induced and five under-expressed. Reduction in transcript levels of genes related to histone is associated to suppression of chromatin organization and DNA methylation (Choi et al., 2015).

AGO4 reduces geminivirus infection by viral DNA methylation (Mallory and Vaucheret, 2010), but this protein may also be recruited by geminiviruses to enhance its transcription (Vinutha et al., 2018). ToLCNDV AC4 protein suppresses RNA silencing by interaction with the host argonaute 4 protein (AGO4) of tomato (Vinutha et al., 2018). Its ortholog protein in melon (MELO3C014440.2) was upregulated at 3 dpi in both susceptible and resistant genotypes.

RNA-dependent RNA polymerase play a key role impairing resistance to geminiviruses, amplifying RNA antiviral silencing (Prakash et al., 2020). In the melon genome, there are eight genes functionally annotated as RDRs and distributed by chromosomes 2, 9, and 10. Two of them were highly upregulated at 6 and 12 dpi in chromosome 9 of PS (MELO3C005284.2 and MELO3C005257.2). An additional one (MELO3C015406.2, chromosome 2) was also induced in this genotype at only 12 dpi. Changes of transcript level in these genes were not observed in the ToLCNDV-resistant genotype WM-7.

Numerous genes coding polymerase enzymes were deregulated in both resistant and susceptible genotypes (Supplementary Tables 5, 6), but a DNA-directed RNA polymerase gene (MELO3C027682.2) was induced at 6 and 12 dpi in the WM-7 genotype.

### Differentially Expressed Genes Validation by qRT-PCR

Seven genes were selected for qRT-PCR validation of the differential response observed in the 28 transcriptomes analysis. Six genes (MELO3C022319.2, MELO3C022313.2, MELO3C022315.2, MELO3C022322.2, MELO3C022327.2, and MELO3C022341.2) were located in the QTL region of chromosome 11 linked to the resistance to ToLCNDV and one gene on chromosome 9 (MELO3C005257.2), and both have been previously described in resistance to other begomoviruses.

**FIGURE 6:**
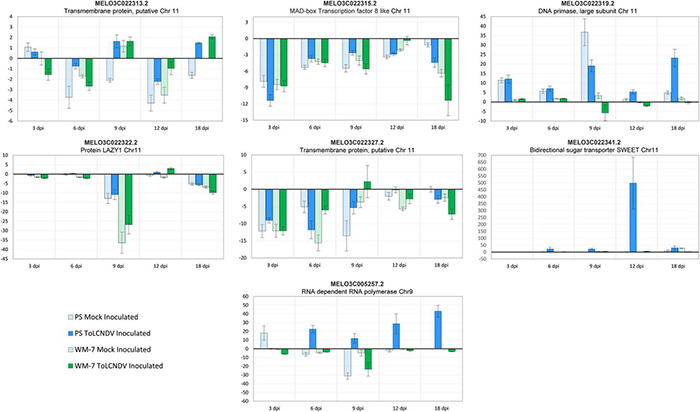
Relative expression 2^(−ΔΔCt)^ of seven candidate genes for ToLCNDV resistance determined by qRT-PCR.

Relative transcript accumulation observed for these genes by qPCR was correlated to the RNA-seq data (Figure 6). These results confirm the high reproducibility between replicates of the transcriptome analysis. MELO3C022327.2 coding a transmembrane protein putatively was the only gene in which low variances were observed between both analysis methodologies. Downregulation of this gene was identified by RNA-seq in only ToLCNDV-inoculated WM-7 samples at 3 and 6 dpi with log2 (fold change) of –1.075 and –3.596, respectively. However, qPCR analysis also detected high downregulation of this same gene in mock-inoculated WM-7 samples at similar levels to virus-inoculated plants. The small transcript level of this gene could be missed in sequencing assay but specifically amplified by qRT-PCR and overrepresented by the 2^−ΔΔCt^ method to determine the relative amount of expression.

### Single Nucleotide Polymorphism Linked to Differentially Expressed Genes Associated With *Tomato Leaf Curl New Delhi Virus* Response

Out of 121,509 total genetic transcriptome-based variant positions found comparing WM-7 and PS, only 31,070 homozygous polymorphisms between both genotypes were considered in this study, excluding common variants (Supplementary Table 11). There were 128 variants located in the major candidate region in chromosome 11, and 951 and 847 were located within the QTL regions with a minor effect in chromosomes 2 and 12, respectively (Supplementary Table 11).

A predicted high impact over genes of the candidate region in chromosome 11 was not associated to any SNP. However, 23 SNPs had a moderate impact on 16 genes, causing a missense change that produces a different amino acid. Three of these SNPs were affecting a GYF domain-containing protein (MELO3C022336.2). A low effect was predicted in 46 SNPs producing in most cases synonymous variants, four of them located in DEGs coding for an Auxin-responsive protein SAUR36 (MELO3C022337.2), a Glutaredoxin protein (MELO3C022339.2), and an ethylene-responsive TF ERF113-like (MELO3C022358.2). In addition, three SNPs with low impact changed a region of the splice site of three genes (MELO3C022343.2 coding a Pyruvate dehydrogenase E1 component subunit beta, MELO3C022352.2 coding a coiled-coil domain-containing protein SCD2, and MELO3C022360.2 coding a Glycosyl transferase). The remaining SNPs identified within this region had a modifier effect, and therefore, their impact is difficult to predict. Four modifier SNPs interestingly affected two DEGs (Supplementary Table 11). In MELO3C022337.2, one SNP was a modifier of the 5′-UTR region. Conversely, in the DNA primase large subunit gene (MELO3C022319.2, down-regulated in WM-7), one SNP affected the 3′-UTR region and a second one had upstream predicted effect. Additional variants in DEGs with downstream effects or in heterozygosity were assumed to have a minimal impact in molecular functionality associated to ToLCNDV response.

In chromosome 2, there were six SNPs with high impact. Three of these variants caused a STOP codon in a 7SK snRNA methylphosphate capping enzyme (MELO3C024707.2) and in two unknown proteins (MELO3C017337.2 and MELO3C029555.2). Furthermore, one splice acceptor and intron variant hit the 5-formyltetrahydrofolate cyclo-ligase (MELO3C017137.2), whereas one insertion caused a frame shift in an unknown protein (MELO3C029754.2). However, the most significant variant conferring a high impact in a DEG of this region (25,433,111 bp) set a mutation of a stop codon into a non-stop codon and, moreover, hit a splice region of the TF bHLH47 (MELO3C017166.2), a gene upregulated in WM-7 at 12 dpi. Between the remaining DEGs of this region, in 40 genes, there were variants with moderate, low, or modifier impact. The upregulated gene in PS MELO3C017364.2 that encodes Ribosomal protein L19 was the DEG affected by the higher number of SNPs (22) in this region, including both missense variants and variants with impact on the 3′-UTR and 5′-UTR.

In the chromosome 12 candidate region, five SNPs had high impact (7,243,329, 9,455,480, 9,746,781, 10,176,940, and 15,255,896 bp), but only one (9,746,781 bp) was located on a DEG coding a splicing factor U2af small subunit B-like (MELO3C004799.2) upregulated in WM-7 at 6 dpi. In addition, this region included 48 DEGs affected by SNPs with moderate, low, or modifier impact, including DEGs described above, such as both DNA-directed RNA polymerases II and IV subunit 5A (MELO3C021758.2) and a Mitogen-activated protein kinase (MELO3C026848.2). It is worth highlighting that 17 SNPs generated different levels of impact on a serine carboxypeptidase-like protein (MELO3C004946.2), repressed in PS at 3 dpi. Moreover, in genes with not altered expression, six variants were within regions of the splice site, and three hit a splice donor or acceptor site, entailing predicted low and high impact, respectively.

## Discussion

ToLCNDV strains generate devastating damage in solanaceaous and cucurbitaceous crops, mainly in the Indian subcontinent and in the Mediterranean basin. Genetic resistance has been previously identified in different species of both horticultural families, but characterization of the molecular mechanism regulating resistance is preferentially studied in *Solanaceaous* crops (Sahu et al., 2010, 2016; Kushwaha et al., 2015; Jeevalatha et al., 2017).

Recessive resistance or incomplete dominance to ToLCNDV is reported as the main inheritance model controlling the resistance in cucurbits (Sáez et al., 2017, 2020, 2021). In this study, we investigate the process of ToLCNDV infection in a resistant and susceptible *C. melo* genotype, trying to correlate resistance or susceptibility with changes in transcript expression patterns and comparing them at different temporal stages. The accession WM-7 is resistant to ToLCNDV, remaining symptomless till 30 days after mechanical inoculation, and this resistance is controlled by a major QTL in chromosome 11 with intermediate dominance and two additional QTLs in chromosomes 2 and 12 (López et al., 2015; Sáez et al., 2017; Romay et al., 2019). Using both approaches, time course infection and quantitative detection of viral load, we observed that virus replication in WM-7 takes place at the beginning of the infection, but its propagation and/or replication is impaired at early stages. Conversely, systemic infection and symptom development progresses in PS with an enhancement of ToLCNDV accumulation in the course of the disease. The behavior observed in WM-7 reflects a fast and time-persistent defense response.

We identified 10 candidate DEGs within the region covering the QTL for ToLCNDV resistance in chromosome 11, most of them associated with a defense response against geminiviruses. The expression of a glutaredoxin protein (MELO3C022339.2) is inhibited in PS at 6 dpi. This gene family is associated with JA-SA pathway (Li et al., 2017c), and moreover, MELO3C022339.2 is an ortholog to the Thioredoxin superfamily proteins of *A. thaliana*. Thioredoxin proteins are described as interacting with begomoviruses and also impairing resistance to potyviruses (Luna-Rivero et al., 2016; Liu et al., 2017; Mathioudakis et al., 2018). In response to TYLCV, thioredoxin and TCP proteins in tomato interact with TFs that regulate defense mechanisms (Huang et al., 2016). TCP TFs are linked to ubiquitination, SUMO, MYC2 pathways, lipooxygenases encoded at chloroplast, and JA biosynthesis and are proposed as responsible candidates for leaf curling during ToLCNDV infection in tomato (Schommer et al., 2008; Naqvi et al., 2010). Although a TCP20-like TF (MELO3C022331.2) is located within the candidate region for ToLCNDV resistance in chromosome 11 of C. melo, we have not identified expression changes during the viral infection course.

Within the same candidate region in chromosome 11, a bidirectional sugar transporter SWEET (MELO3C022341.2) is the gene with higher induction in the susceptible response of PS at 12 dpi. SWEET transporters facilitate sugar transference into the phloem and promote their transport (Chen, 2014). Some studies report transcription changes of SWEET genes in several crops after pathogens attack, including plant–begomovirus interactions (Antony et al., 2010; Chen, 2014; Chandran, 2015; Naqvi et al., 2017; Breia et al., 2020). In melon, plants infected with CMV accumulated more sugars in the phloem of leaves as result of the sucrose transporter effect (Gil et al., 2011). In cotton (*Gossypium* spp.), a SWEET transporter gene was downregulated in resistant plants after *cotton leaf curl disease* (CLCuD) infection (Naqvi et al., 2017), and involvement of sugar-signaling mechanisms has been observed in resistance plants to TYLCV (Sade et al., 2020). Out of the 24 genes coding for SWEET transporters in the *C. melo* genome, three DEGs of PS were located outside the QTL regions: on chromosome 12, MELO3C001650.2 was repressed at 12 dpi, whereas MELO3C005869.2 and MELO3C002381.2 were upregulated at 6 and 12 dpi in chromosomes 9 and 12, respectively.

Another DEG identified in this region encodes a DNA primase large subunit (MELO3C022319.2), which is downregulated in WM-7 after ToLCNDV inoculation, whereas in PS, it is induced. Interestingly, this gene is syntenic with the DNA primase gene in chromosome 8 of *Cucurbita moschata*, in which a mayor QTL controlling resistance to ToLCNDV has been identified (Sáez et al., 2020). Indeed, this is the only DEG shared between both syntenic regions in which we have also identified genetic polymorphisms between WM-7 and PS. Geminiviruses employ the host mechanism for triggering their own replication. Plant DNA primases are described as catalyst enzymes used in the first step of geminivirus replication (Saunders et al., 1992; Alberter et al., 2005). Thus, the reduced expression of MELO3C022319.2 in WM-7 likely contributes to disrupting transcription of ToLCNDV, which appears as a promising mechanism of resistance, developing a downstream defense response.

It is worth mentioning that the candidate region on chromosome 12 includes an NAC TF (MELO3C004694.2), highly induced in PS at 12 dpi. The same induction is reported for two NAC TF membranes upon ToLCNDV infection in tomato (Bhattacharjee et al., 2017) and, in prior works, Román et al. (2019) validates by qPCR a, NAC TF highly induced in PS accession. ToLCNDV accumulation and symptom severity seem to correlate with the expression of the NAC TF.

Most of the recessive genes involved in resistance to RNA plant viruses codify for eukaryotic translation initiation factors eIF4E/G, a protein complex that mediates recruitment of ribosomes, in which natural mutations lead to resistance by loss of function (Kachroo et al., 2017; Machado et al., 2017). In the case of begomoviruses, six *loci* are described conferring resistance to *Tomato yellow leaf curl virus* (TYLCV) in tomato, one has a recessive inheritance pattern (*ty-5*) (Anbinder et al., 2009; Hutton et al., 2012), and two of them have incomplete dominant control (*Ty-4* and *Ty-6*) (Yan et al., 2021). The *ty*-*5 locus* causes resistance by the loss of function of the messenger RNA surveillance factor Pelota (*Pelo*), which is involved in biosynthesis of ribosomes, and its dysfunction compromises the translational machinery and replication of begomoviruses (Lapidot et al., 2015). This gene is also reported in *Capsicum annuum* conferring resistance to mono and bipartite begomoviruses (Prins et al., 2019; Koeda et al., 2021). Furthermore, NIK1 (NSP-interacting kinase 1) or RIP (Ribosome-inactivating) proteins are associated with the prevention of the spread of geminiviruses through translational suppression or repression of components of the translational machinery, such as ribosomes (Hong et al., 1996; Zorzatto et al., 2015; Musidlak et al., 2017; Citores et al., 2021). In contrast to what was expected, changes in the transcript level orthologous to these genes in melon after ToLCNDV infection were not identified in this study. However, an important number of genes related to ribosomal pathways were downregulated in WM-7. Moreover, the minor region modulating the response to ToLCNDV in chromosome 2 of *C. melo* includes a Ribosomal protein L19 (MELO3C017364.2) remarkably affected by a high number of SNPs, upregulated at 6 and 12 dpi in PS. As viruses require host enzymes for translation (Ignacio-Espinoza et al., 2020; Dong et al., 2021; Xiong et al., 2021), evolutionarily conserved genes involved in ribosome recruitment might be a plausible mechanism triggering the resistance response to ToLCNDV in melon.

Prior works suggest an interconnection between loss of function of translation factors and replication of the viral genomes, changes in the cell wall affecting viral movement and activation of immunity pathways (Sanfaçon, 2015; Ding et al., 2018). Indeed, geminiviruses not only recruit plant translational components, but also enhance their replication and spread using and relocalizing host genes involved in DNA replication and transcription (Preiss and Jeske, 2003; Kushwaha et al., 2017; Wu et al., 2019; Maio et al., 2020). The interference with this viral strategy is widely described conferring resistance to begomoviruses (Ullah et al., 2014; Chakraborty and Basak, 2018). In tomato, both 26S proteasomal subunit RPT4a (*SlRPT4*) and DEAD-box RNA helicase genes are described as interfering in ToLCNDV transcription and replication, respectively (Sahu et al., 2010, 2016; Pandey et al., 2019). In this study, none of their orthologs in melon showed differences in their transcription upon ToLCNDV infection. However, genes belonging to the RNA helicase gene family have altered expression in PS at all analyzed stages, and GO ontologies related to DNA conformation, replication, unwinding, cell cycle, DNA helicase activity, and MCM complex were enriched, including upregulated genes in PS that were repressed in WM-7. DNA helicases are required for strand separation during DNA replication. Their analogs, RNA helicases, are described as proteins hijacked by plant viruses to assist their replication (Ranji and Boris-Lawrie, 2010; Sharma and Boris-Lawrie, 2012). Although little research about host DNA helicases and geminiviruses has been reported, interaction between geminiviral proteins and DNA helicases is described as assisting DNA replication (Pradhan et al., 2017). Host DNA helicase proteins can be used by the virus to further strengthen the helicase activity of the geminiviral Rep protein and, therefore, generate unwinding and initiate the stem-loop formation on the viral DNA (Brister and Muzyczka, 1999; Kazlauskas et al., 2016). Furthermore, the MCM complex has a role in maintaining the minichromosome and is directly implicated with the initiation of DNA synthesis at replication origins (Forsburg, 2004; Bochman and Schwacha, 2008). There are reports pointing out the role of the MCM2 protein in geminivirus replication efficiency (Rizvi et al., 2015), but molecular mechanisms remain unknown (Cho et al., 2008; Suyal et al., 2013). Overall, induced DNA replication transcriptional pathways in PS are likely promoting ToLCNDV multiplication and systemic propagation.

To further analyze the changes of expression patterns regarding plant pathogen interaction between melon and ToLCNDV, we studied defense genes and pathways activated in similar pathosystems. In a recent work (Sharma et al., 2021), the *Sw5a* locus is reported as an R gene that recognizes ToLCNDV AC4 protein and restricts viral spread. Although the melon ortholog to this gene (MELO3C006780.2) is not differentially expressed in our system, it appears positively correlated to PS in CG19 and CG20, indicating that defense responses against ToLCNDV are not efficiently activated in this genotype. In addition, we identified other DEGs implicated in virus resistance distributed over the melon genome, most of them located in clusters at chromosomes 1, 5, and 9. The two largest NBS-LRR gene clusters in melon are located at chromosomes 5 and 9, where resistance to other cucurbit viruses has been mapped (Morata and Puigdomènech, 2017; Pérez-de-Castro et al., 2020). Chromosome 5 not only includes differentially regulated pathogenesis and R genes, but also lipoxygenase hormone biosynthesis genes induced in WM-7 and downregulated in PS. These hormones regulate cell death and JA biosynthetic routes and conduct lipid peroxidation as a response to pathogen infection (Hwang and Hwang, 2010). Plant–virus interaction studies evidence how geminiviruses regulate JA signaling, hijacking implicated genes and increasing susceptibility (Yan and Xie, 2015; Zhang et al., 2017; Guerrero et al., 2020). In this work, we identify transcriptional changes in JA genes required for resistance to viruses. MYC-2 TF (MELO3C022250.2), mitogen-activated protein kinases (MELO3C026848.2), and the terpene cyclase/mutase gene family (MELO3C022374.2) are downregulated in the susceptible genotype PS at the beginning of infection (3 and 6 dpi). Conversely, some of these genes are induced in WM-7 at same stages. In tomato (*Solanum lycopersicum*), induction of a mitogen-activated protein kinase 3 (*SlMPK3*) ortholog to MELO3C026848.2 here identified enhanced TYLCV resistance by an increase of SA/JA-gene expression as PR-1 or leucine aminopeptidases (Li et al., 2017b). Leucine aminopeptidases control the defense machinery in tomato, downstream of JA (Fowler et al., 2009). Here, we identify one gene (MELO3C004135.2) codifying this protein located on chromosome 5 of *C. melo*, highly induced in WM-7 at 3 dpi but downregulated in PS at 6 dpi (Supplementary Table 7). Additionally, we observed differential regulation at early stages of other genes, such as E3 ligases, LAF 1, and TFs participating in ubiquitination and photomorphogenesis, all interconnected with jasmonic signaling (Seo et al., 2001; Kazan and Manners, 2011; Lozano-Durán et al., 2011; Correa et al., 2013). Viral replication, cell-to-cell movement, and long-distance propagation are inhibited by SA and JA (Shang et al., 2011; Tsai et al., 2019). Thus, implication of all described genes in the JA pathway indicates either an interference or perhaps a recruitment of this route by ToLCNDV in PS as a strategy to increase the susceptible response at early stages. Instead, the opposite response shown over this hormonal pathway in WM-7 likely promotes resistance.

In chromosome 9, where the second larger R-gene cluster is located, two RDR genes were over-expressed in PS (MELO3C005284.2 and MELO3C005257.2) at 6 and 12 dpi. Those genes are orthologs to RDR1 (RDRα) of *Arabidopsis thaliana*, involved in amplification of the gene silencing signal at the post-transcriptional level in virus infection (Qui et al., 2009; Willmann et al., 2011; Islam et al., 2018). RDRs are well described in the RNA silencing pathway, and their over-expression enhances resistance to begomoviruses, particularly to those of the tomato leaf curl viruses complex (Prakash et al., 2020). This function of RDR1 in geminivirus resistance is variable and depends on the virus species (Chen et al., 2010; Aregger et al., 2012). In *N. tabacum*, RDR1 is associated with recovery *tomato leaf curl Gujarat virus* (ToLCGV) infected plants, generating hypermethylation of the viral genome. However, in those *N. tabacum* plants infected with ToLCNDV, recovery is inhibited by AV2 viral protein, resulting in susceptible plants with high symptomatology and viral titers (Basu et al., 2018). In *N. benthamiana* a natural mutation of 72 bp insertion in the RDR1 gene promotes susceptibility to members of the *Tobamovirus* genus (Yang et al., 2004) and might be responsible for high susceptibility to numerous viruses observed in this species, including geminiviruses (Akhtar et al., 2011). In cucumber (*Cucumis sativus* L.), the RDR1 family has four subclasses (RDR1a, RDR1b, RDR1c1, RDR1c2). RDR1c1 and RDR1c2 are highly induced by geminivirus *squash leaf curl virus* (SLCV) in susceptible plants, but their expression level remains low in resistant plants (Leibman et al., 2018). Also, in *C. sativus*, RDR1 is notably induced by a viroid infection, suggesting the involvement of RDR1 in the antiviroid defense (Xia et al., 2017). Our results concerning RDR1 expression in resistant and susceptible melon genotypes follows a similar expression trend with very high expression in PS but unaltered in WM-7. However, polymorphic variations have not been identified in this work affecting any of these both genes.

A third RDR gene (MELO3C015406.2) was upregulated in PS in chromosome 2 at only 12 dpi. It is a RDR5 protein with a homologous sequence to the *Ty1/Ty3* gene conferring resistance to TYLCV by methylation of cytosines in the viral genome (Verlaan et al., 2013; Butterbach et al., 2014; Jackel et al., 2016; Gallego-Bartolomé et al., 2019). Despite their homology, MELO3C015406.2 was over-expressed in the susceptible genotype to ToLCNDV, conversely to what was expected, as *Ty1* expression is elevated in resistant lines (Verlaan et al., 2013). Tomato cultivars carrying *Ty1/Ty3* genes are described as displaying resistant behavior after ToLCNDV inoculation (Fortes et al., 2016; Hussain et al., 2019). However, the genetic background effect might modulate the effectiveness of *Ty1/Ty3* for resistance to ToLCNDV in tomato (Rai et al., 2013; Akhtar et al., 2019). In this work, we identify two SNPs generating missense variations in MELO3C015406.2, but QTLs linked to ToLCNDV resistance have not been mapped covering this region in previous works. Results obtained here suggest that infection of ToLCNDV induces and triggers the gene-silencing machinery in PS, but it is not efficient in controlling virus spread. Another approach to explain this behavior is that RDRs are targeted and induced by ToLCNDV, interfering with silencing mechanism for defense and deregulating host factors, which result in symptomatology display. Further studies must be conducted to characterize this process in begomovirus–cucurbits interaction.

In TGS, DNA methylation plays crucial role inhibiting viral DNA transcription and replication. Plant hosts with this pathway suppressed are strongly susceptible to geminiviruses (Raja et al., 2014). Besides RDRs, other genes involved in RNA silencing–based resistance to geminiviruses are identified with altered expression in this study, including histones, calmodulin proteins, AGO4, and methyltransferases. MET1 and CMT3 DNA methyltransferases are induced at early stages in WM-7 and repressed in PS after ToLCNDV inoculation. In geminivirus infection, Rep protein interacts with CMT3 and MET1 in *N. benthamiana* and *A. thaliana*, suppressing their expression and the maintenance of DNA methylation (Rodríguez-Negrete et al., 2013). A phosphoethanolamine *N*-methyltransferase (MELO3C017356.2) was upregulated in PS at early stages in the candidate region of chromosome 2. This protein has homology with the *S*-adenosyl-L-methionine-dependent methyltransferases of *A. thaliana.* Methylation of DNA is performed by cellular methyltransferases, using *S*-adenosyl methionine (SAM). *Arabidopsis* plants with mutated genes of SAM enzyme production are hypersensitive to geminiviruses (Mäkinen and De, 2019). *Beet severe curly top virus* (BSCTV) C2 protein interacts and inhibits SAM by decarboxylation, reducing methylation of the viral DNA and promoting infection (Zhang et al., 2011). C4 protein of *cotton leaf curl multan virus* (CLCuMuV) interacts with SAMS of *N. benthamiana*, reducing its activity and DNA methylation and promoting accumulation of CLCuMuV (Ismayil et al., 2018). Expression changes in PS of this kind of enzyme suggests its interaction with ToLCNDV even in susceptible response. DNA-directed RNA polymerases are components of the RNA-directed DNA methylation (RdDM) epigenetic process, mediating geminivirus silencing and conferring resistance (Jackel et al., 2016). Although several genes of this family are differentially expressed in both PS and WM-7 (Supplementary Tables 5, 6), three DNA-directed RNA polymerases coding genes (MELO3C027682.2, MELO3C000330.2, and MELO3C028015.2 clustering together on chromosome 7) are highly induced only in WM-7 at 6 or 12 dpi.

Cell-to-cell movement and viral replication, encapsidation, and suppression of host defenses are essential steps in the infective cycle of plant viruses, and both are connected by means of cellular membranes (Levy and Tilsner, 2020). Components of membrane, membrane and transport activity GO categories were found enriched in DEGs in both susceptible and resistance genotypes. Interestingly, genes annotated in GO category “Thylakoid” GO in GC20. Induction of the chloroplast thylakoid membrane protein TMP14 in *N. benthamiana* increased disease symptom severity and virus accumulation of *Tomato Spotted Wilt Virus* (TSWV), evidencing that lack of this protein negatively affects viral infection (Zhan et al., 2021). In WM-7, the GO category “photosynthesis” was the most represented of the upregulated genes at 6 and 12 dpi. In plant–virus interactions, the chloroplast is strategically manipulated and damaged by viruses, affecting large proportions of genes (Bhattacharyya and Chakraborty, 2018; Rossitto de Marchi et al., 2020; Zhai et al., 2020).

Chlorosis and mosaics in *cucumber mosaic virus* (CMV) infected tobacco leaves (*Nicotiana tabacum*) are associated with downregulation of photosynthetic and chloroplastic genes (Mochizuki et al., 2014), and similar genes had a reduced transcription in chlorotic tissues of watermelon [*Citrullus lanatus* (Thunb.) Matsum. & Nakai] systemically infected with *Cucumber green mottle mosaic virus* (CGMMV) (Sun et al., 2019). Among begomoviruses, transcriptional reprogramming caused by TYLCV infection in *N. benthamiana* generated an expression reduction of photosynthesis genetic pathways (Wu et al., 2019). Plant defense against virus infection usually involves high energetic costs for the host, for example, for antiviral RNA silencing. Consequently, multiple and complementary resistance mechanisms are activated to minimize this energy consumption (Souza et al., 2019). The high induction of chloroplastic and photosynthetic genes detected in WM-7 takes place at the stages when yellowing and mosaic symptoms are developed in PS. These genes could provide additional energy to the resistant plant cells to address the viral attack and avoid chloroplast damage and symptom development. Moreover, a repression of genes implicated with thylakoid membranes in WM-7 may indicate an additional obstacle to ToLCNDV to reach the photosynthetic machinery and components, increasing the resistance to this virus and protecting fruit yield in melon.

## Conclusion

The data reported in this study demonstrate that ToLCNDV infection entails a complex and interconnected net of transcriptional rearrangements in melon. Our findings advance the molecular understanding underlying ToLCNDV infection in melon. Combining both DEGs and polymorphic SNP detection, we identify candidate genes linked to the three QTLs for resistance in WM-7. The lack or decrease in ToLCNDV replication is associated with the most promising candidate in chromosome 11, a DNA primase protein. Whereas in minor modifier regions of chromosomes 2 and 12, some of the described bHLH47 or NAC TFs, Mitogen-activated kinase, DNA-directed RNA polymerases, or alternative splicing events likely might act as potential factors triggering a transcriptomic cascade of genetic responses against viral infection. Although promising, further studies based on genome edition or induced gene silencing are required to functionally characterize these target genes.

## Data Availability Statement

The datasets presented in this study can be found in online repositories. The names of the repository/repositories and accession number(s) can be found below: NCBI SRA, PRJNA780428.

## Author Contributions

CS, BP, and CL conceived and designed the research. CS, CL, and AS performed the tests with ToLCNDV. CS, AF-L, and JM-P performed the bioinformatics analysis. CS and AS conducted the qPCR validation analysis. CS, CL, and BP conducted and wrote the manuscript with important contributions from JM-P, ND, and AS. All authors read and approved the final manuscript.

## Conflict of Interest

The authors declare that the research was conducted in the absence of any commercial or financial relationships that could be construed as a potential conflict of interest.

## Publisher’s Note

All claims expressed in this article are solely those of the authors and do not necessarily represent those of their affiliated organizations, or those of the publisher, the editors and the reviewers. Any product that may be evaluated in this article, or claim that may be made by its manufacturer, is not guaranteed or endorsed by the publisher.

## Footnotes

1
http://cucurbitgenomics.org/

## Supplementary Material

Supplementary Figure 1 Gene expression patterns of the analyzed samples for each gene cluster. Eigengene values are represented across tissues.

Supplementary Figure 2 Scatterplot showing grouped GO terms according to the most representative categories. Figures were generated using the REVIGO program loading the list of enriched GO terms of WM-7 and PS separately. Positive and negative numbers in both axes represent upregulated and downregulated genes, respectively.

Supplementary Table 1 Primer sequences used in qPCR for DEG confirmation assay.

Supplementary Table 2 Genes significantly correlated to each gene cluster (GC) at *p*-value < 0.01. Their functional description and ontology annotation are presented. Those genes with unavailable GO terms associated are shown as NA.

Supplementary Table 3 GO terms and KEGG enrichments of the 21 gene clusters (GCs). KEGG and GO ontologies (BP, biological processes; MF, molecular function; CC, cellular component) include genes significantly correlated at *p*-value < 0.01.

Supplementary Table 4 Significance (*p*-value) of each gene cluster (GC) after regression analyses using genotype, treatment, and time as factors.

Supplementary Table 5 DEGs in WM-7 at 3, 6, and 12 dpi in ToLCNDV and mock-inoculated plants. Log2 (fold change) values, gene code, its genomic position in melon genome (v4.0) and functional annotation are shown.

Supplementary Table 6 DEGs in PS at 3, 6, and 12 dpi in ToLCNDV and mock-inoculated plants. Log2 (fold change) values, gene code, its genomic position in melon genome (v4.0) and functional annotation are shown.

Supplementary Table 7 Common DEGs upregulated in WM-7 and downregulated in PS at 3, 6, and 12 dpi. Log2 (fold change) values, gene code, its genomic position in melon genome (v4.0) and functional annotation are shown.

Supplementary Table 8 Common DEGs at different stages (3, 6, and 12 dpi) after ToLCNDV inoculation in WM-7 genotype.

Supplementary Table 9 Common DEGs at different stages (3, 6, and 12 dpi) after ToLCNDV inoculation in PS genotype.

Supplementary Table 10 DEGs at 3, 6, and 12 dpi in the candidate regions of chromosomes 2 and 12 of PS and WM-7, conferring resistance to ToLCNDV.

Supplementary Table 11 List of polymorphic transcript-based SNPs between WM-7 and PS with both alleles fixed in homozygosity. SNP position, reference and alternative alleles, genotype for each sample, affected genes, and putative impact of the SNP on the gene structure are shown. The reference allele is represented as 0 and the alternative allele as 1. Cells highlighted in yellow refer to DEGs. Horizontal lines in chromosomes 2, 11, and 12 delimit candidate regions considered in this study for ToLCNDV resistance.

## References

[B1] Akhtar K. P. Akram A. Ullah N. Saleem M. Y. Saeed M. (2019). Evaluation of *Solanum* species for resistance to tomato leaf curl New Delhi virus using chip grafting assay. *Sci. Hortic.* 256:108646. 10.1016/j.scienta.2019.108646

[B2] Akhtar S. Briddon R. W. Mansoor S. (2011). Reactions of *Nicotiana* species to inoculation with monopartite and bipartite begomoviruses. *Virol. J.* 8:475. 10.1186/1743-422X-8-475 22011413 PMC3213157

[B3] Alberter B. Rezaian M. A. Jeske H. (2005). Replicative intermediates of tomato leaf curl virus and its satellite DNAs. *Virology* 331 441–448. 10.1016/j.virol.2004.10.043 15629786

[B4] Allie F. Pierce E. J. Okoniewski M. J. Rey M. E. C. (2014). Transcriptional analysis of South African cassava mosaic virus-infected susceptible and tolerant landraces of cassava highlights differences in resistance, basal defense and cell wall associated genes during infection. *BMC Genomics* 15:1006. 10.1186/1471-2164-15-1006 25412561 PMC4253015

[B5] Allie F. Rey M. E. C. (2013). Transcriptional alterations in model host, *Nicotiana benthamiana*, in response to infection by south African cassava mosaic virus. *Eur. J. Plant Pathol.* 137 765–785. 10.1007/s10658-013-0286-4

[B6] Anbinder I. Reuveni M. Azari R. Paran I. Nahon S. Shlomo H. (2009). Molecular dissection of tomato leaf curl virus resistance in tomato line TY172 derived from *Solanum peruvianum*. *Theor. Appl. Genet.* 119 519–530. 10.1007/s00122-009-1060-z 19455299

[B7] Andrews S. (2010). *FastQC: A Quality Control Tool for High Throughput Sequence Data.* Available online at: http://www.bioinformatics.babraham.ac.uk/projects/fastqc (accessed September 15, 2018).

[B8] Annu P. K. Rani R. Rathi A. S. (2019). Gemini viruses-emerging threat to crops. *J. Pharmacogn. Phytochem.* 8 2006–2012.

[B9] Antony G. Zhou J. Huang S. Li T. Liu B. White F. (2010). Rice xa13 recessive resistance to bacterial blight is defeated by induction of the disease susceptibility gene Os-11N3. *Plant Cell* 22 3864–3876. 10.1105/tpc.110.078964 21098734 PMC3015117

[B10] Aregger M. Borah B. K. Seguin J. Rajeswaran R. Gubaeva E. G. Zvereva A. S. (2012). Primary and secondary siRNAs in geminivirus-induced gene silencing. *PLoS Pathog.* 8:e1002941. 10.1371/journal.ppat.1002941 23028332 PMC3460622

[B11] Balint-Kurti P. (2019). The plant hypersensitive response: concepts, control and consequences. *Mol. Plant Pathol.* 20 1163–1178. 10.1111/mpp.12821 31305008 PMC6640183

[B12] Bardou P. Mariette J. Escudié F. Djemiel C. Klopp C. (2014). jvenn: an interactive Venn diagram viewer. *BMC Bioinformatics* 15:293. 10.1186/1471-2105-15-293 25176396 PMC4261873

[B13] Basu S. Kumar Kushwaha N. Kumar Singh A. Pankaj Sahu P. Vinoth Kumar R. Chakraborty S. (2018). Dynamics of a geminivirus-encoded pre-coat protein and host RNA-dependent RNA polymerase 1 in regulating symptom recovery in tobacco. *J. Exp. Bot.* 69 2085–2102. 10.1093/jxb/ery043 29432546 PMC6019014

[B14] Bhattacharjee P. Das R. Mandal A. Kundu P. (2017). Functional characterization of tomato membrane-bound NAC transcription factors. *Plant Mol. Biol.* 93 511–532. 10.1007/s11103-016-0579-z 28039561

[B15] Bhattacharyya D. Chakraborty S. (2018). Chloroplast: the Trojan horse in plant-virus interaction. *Mol. Plant Pathol.* 19 504–518. 10.1111/mpp.12533 28056496 PMC6638057

[B16] Bochman M. L. Schwacha A. (2008). The Mcm2-7 complex has in vitro helicase activity. *Mol. Cell* 31 287–293. 10.1016/j.molcel.2008.05.020 18657510

[B17] Bolger A. M. Lohse M. Usadel B. (2014). Trimmomatic: a flexible trimmer for Illumina sequence data. *Bioinformatics* 30 2114–2120. 10.1093/bioinformatics/btu170 24695404 PMC4103590

[B18] Bonza M. C. Fusca T. Homann U. Thiel G. De Michelis M. I. (2009). Intracellular localisation of PPI1 (proton pump interactor, isoform 1), a regulatory protein of the plasma membrane H^+^-ATPase of *Arabidopsis thaliana*. *Plant Biol.* 11 869–877. 10.1111/j.1438-8677.2008.00181.x 19796364

[B19] Breia R. Conde A. Pimentel D. Conde C. Fortes A. M. Granell A. (2020). VvSWEET7 is a mono- and disaccharide transporter up-regulated in response to *Botrytis cinerea* infection in grape berries. *Front. Plant Sci.* 10:1753. 10.3389/fpls.2019.01753 32047506 PMC6996298

[B20] Brister J. R. Muzyczka N. (1999). Rep-mediated nicking of the adeno-associated virus origin requires two biochemical activities, DNA helicase activity and transesterification. *J. Virol.* 73 9325–9336. 10.1128/JVI.73.11.9325-9336.1999 10516041 PMC112967

[B21] Butterbach P. Verlaan M. G. Dullemans A. Lohuis D. Visser R. G. Bai Y. (2014). Tomato yellow leaf curl virus resistance by *Ty-1* involves increased cytosine methylation of viral genomes and is compromised by cucumber mosaic virus infection. *Proc. Natl. Acad. Sci. U.S.A.* 111 12942–12947. 10.1073/pnas.1400894111 25136118 PMC4156758

[B22] Castanera R. Ruggieri V. Pujol M. García-Mas J. Casacuberta J. M. (2020). An improved melon reference genome with single-molecule sequencing uncovers a recent burst of transposable elements with potential impact on genes. *Front. Plant Sci.* 10:1815. 10.3389/fpls.2019.01815 32076428 PMC7006604

[B23] Castillo-González C. Liu X. Huang C. Zhao C. Ma Z. Hu T. (2015). Geminivirus-encoded TrAP suppressor inhibits the histone methyltransferase SUVH4/KYP to counter host defense. *eLife* 4:e06671. 10.7554/eLife.06671 26344546 PMC4606454

[B24] Chakraborty N. Basak J. (2018). Molecular and biochemical characterization of mungbean yellow mosaic India virus resistance in leguminous host *Vigna mungo*. *J. Plant Biochem. Biotechnol.* 27 318–330. 10.1007/s13562-018-0441-2

[B25] Chandran D. (2015). Co-option of developmentally regulated plant SWEET transporters for pathogen nutrition and abiotic stress tolerance. *IUBMB Life* 67 461–471. 10.1002/iub.1394 26179993

[B26] Chen H. Tamai A. Mori M. Ugaki M. Tanaka Y. Samadder P. P. (2010). Analysis of rice RNA-dependent RNA polymerase 1 (OsRDR1) in virus-mediated RNA silencing after particle bombardment. *J. Plant Pathol.* 76 152–160. 10.1007/s10327-010-0226-5

[B27] Chen L. Q. (2014). SWEET sugar transporters for phloem transport and pathogen nutrition. *New Phytol.* 201 1150–1155. 10.1111/nph.12445 24649486

[B28] Cho J. H. Kim H. B. Kim H. S. Choi S. B. (2008). Identification and characterization of a rice MCM2 homologue required for DNA replication. *BMB Rep.* 41 581–586. 10.5483/bmbrep.2008.41.8.581 18755073

[B29] Choi H. Jo Y. Lian S. Jo K. M. Chu H. Yoon J. Y. (2015). Comparative analysis of chrysanthemum transcriptome in response to three RNA viruses: cucumber mosaic virus, tomato spotted wilt virus and potato virus X. *Plant Mol. Biol.* 88 233–248. 10.1007/s11103-015-0317-y 25904110

[B30] Chung H. Y. Lacatus G. Sunter G. (2014). Geminivirus AL2 protein induces expression of, and interacts with, a calmodulin-like gene, an endogenous regulator of gene silencing. *Virology* 460–461 108–118. 10.1016/j.virol.2014.04.034 25010276

[B31] Cingolani P. Platts A. Wang L. L. Coon M. Nguyen T. Wang L. (2012). A program for annotating and predicting the effects of single nucleotide polymorphisms, SnpEff. *Fly* 6 80–92. 10.4161/fly.19695 22728672 PMC3679285

[B32] Citores L. Iglesias R. Ferreras J. M. (2021). Antiviral activity of ribosome-inactivating proteins. *Toxins* 13:80. 10.3390/toxins13020080 33499086 PMC7912582

[B33] Conesa A. Madrigal P. Tarazona S. Gomez-Cabrero D. Cervera A. McPherson A. (2016). A survey of best practices for RNA-seq data analysis. *Genome Biol.* 17:13. 10.1186/s13059-016-0881-8 26813401 PMC4728800

[B34] Correa R. L. Bruckner F. P. de Souza Cascardo R. Alfenas-Zerbini P. (2013). The role of F-box proteins during viral infection. *Int. J. Mol. Sci.* 14 4030–4049. 10.3390/ijms14024030 23429191 PMC3588083

[B35] Czosnek H. Eybishtz A. Sade D. Gorovits R. Sobol I. Bejarano E. (2013). Discovering host genes involved in the infection by the tomato yellow leaf curl virus complex and in the establishment of resistance to the virus using tobacco rattle virus-based post transcriptional gene silencing. *Viruses* 5 998–1022. 10.3390/v5030998 23524390 PMC3705308

[B36] Ding W. Wu J. Ye J. Zheng W. Wang S. Zhu X. (2018). A Pelota-like gene regulates root development and defence responses in rice. *Ann. Bot.* 122 359–371. 10.1093/aob/mcy075 29771278 PMC6110353

[B37] Dobin A. Davis C. A. Schlesinger F. Drenkow J. Zaleski C. Jha S. (2013). STAR: ultrafast universal RNA-seq aligner. *Bioinformatics* 29 15–21. 10.1093/bioinformatics/bts635 23104886 PMC3530905

[B38] Dong H. J. Zhang R. Kuang Y. Wang X. J. (2021). Selective regulation in ribosome biogenesis and protein production for efficient viral translation. *Arch. Microbiol.* 203 1021–1032. 10.1007/s00203-020-02094-5 33124672 PMC7594972

[B39] Doyle J. J. Doyle J. L. (1990). Isolation of plant DNA from fresh tissue. *Focus* 12 13–15.

[B40] Forsburg S. L. (2004). Eukaryotic MCM proteins: beyond replication initiation. *Microbiol. Mol. Biol. Rev.* 68 109–131. 10.1128/mmbr.68.1.109-131.2004 15007098 PMC362110

[B41] Fortes I. M. Sánchez-Campos S. Fiallo-Olivé E. Díaz-Pendón J. A. Navas-Castillo J. Moriones E. (2016). A novel strain of tomato leaf curl New Delhi virus has spread to the Mediterranean basin. *Viruses* 8:307. 10.3390/v8110307 27834936 PMC5127021

[B42] Fowler J. H. Narváez-Vásquez J. Aromdee D. N. Pautot V. Holzer F. M. Walling L. L. (2009). Leucine aminopeptidase regulates defense and wound signaling in tomato downstream of jasmonic acid. *Plant Cell* 21 1239–1251. 10.1105/tpc.108.065029 19376935 PMC2685619

[B43] Gallego-Bartolomé J. Liu W. Kuo P. H. Feng S. Ghoshal B. Gardiner J. (2019). Co-targeting RNA polymerases IV and V promotes efficient de novo DNA methylation in *Arabidopsis*. *Cell* 176 1068–1082.e19. 10.1016/j.cell.2019.01.029 30739798 PMC6386582

[B44] Garrison E. Marth G. (2012). Haplotype-based variant detection from short-read sequencing. *arXiv* [Preprint]. arXiv:1207.3907

[B45] Gholizadeh A. Santha I. M. Kohnehrouz B. B. Lodha M. L. Kapoor H. C. (2005). Cystatins may confer viral resistance in plants by inhibition of a virus-induced cell death phenomenon in which cysteine proteinases are active: cloning and molecular characterization of a cDNA encoding cysteine-proteinase inhibitor (celostatin) from *Celosia cristata* (crested cock’s comb). *Biotechnol. Appl. Biochem.* 42 197–204. 10.1042/BA20050029 15842197

[B46] Giacomini D. A. Gaines T. Beffa R. Tranel P. J. (2018). Optimizing RNA-seq studies to investigate herbicide resistance. *Pest Manag. Sci.* 74 2260–2264. 10.1002/ps.4822 29222921

[B47] Gil L. Yaron I. Shalitin D. Sauer N. Turgeon R. Wolf S. (2011). Sucrose transporter plays a role in phloem loading in CMV-infected melon plants that are defined as symplastic loaders. *Plant J.* 66 366–374. 10.1111/j.1365-313X.2011.04498.x 21241389

[B48] Góngora-Castillo E. Ibarra-Laclette E. Trejo-Saavedra D. L. Rivera-Bustamante R. F. (2012). Transcriptome analysis of symptomatic and recovered leaves of geminivirus-infected pepper (*Capsicum annuum*). *Virol. J.* 9:295. 10.1186/1743-422X-9-295 23185982 PMC3546870

[B49] González-Ibeas D. Blanca J. Roig C. González-To M. Picó B. Truniger V. (2007). MELOGEN: an EST database for melon functional genomics. *BMC Genomics* 8:306. 10.1186/1471-2164-8-306 17767721 PMC2034596

[B50] Gorovits R. Czosnek H. (2017). The involvement of heat shock proteins in the establishment of tomato yellow leaf curl virus infection. *Front. Plant Sci.* 8:355. 10.3389/fpls.2017.00355 28360921 PMC5352662

[B51] Gorovits R. Moshe A. Ghanim M. Czosnek H. (2013). Recruitment of the host plant heat shock protein 70 by tomato yellow leaf curl virus coat protein is required for virus infection. *PLoS One* 8:e70280. 10.1371/journal.pone.0070280 23894631 PMC3720902

[B52] Guerrero J. Regedanz E. Lu L. Ruan J. Bisaro D. M. Sunter G. (2020). Manipulation of the plant host by the geminivirus AC2/C2 protein, a central player in the infection cycle. *Front. Plant Sci.* 11:591. 10.3389/fpls.2020.00591 32508858 PMC7248346

[B53] Guilliam T. A. Keen B. A. Brissett N. C. Doherty A. J. (2015). Primase-polymerases are a functionally diverse superfamily of replication and repair enzymes. *Nucleic Acids Res.* 43 6651–6664. 10.1093/nar/gkv625 26109351 PMC4538821

[B54] Gutierrez C. (2000). DNA replication and cell cycle in plants: learning from geminiviruses. *EMBO J.* 19 792–799. 10.1093/emboj/19.5.792 10698921 PMC305619

[B55] Gutiérrez-Campos R. Torres-Acosta J. A. Saucedo-Arias L. J. Gomez-Lim M. A. (1999). The use of cysteine proteinase inhibitors to engineer resistance against potyviruses in transgenic tobacco plants. *Nat. Biotechnol.* 17 1223–1226. 10.1038/70781 10585723

[B56] Hong Y. Saunders K. Hartley M. R. Stanley J. (1996). Resistance to geminivirus infection by virus-induced expression of dianthin in transgenic plants. *Virology* 220 119–127. 8659104 10.1006/viro.1996.0292

[B57] Huang Y. Zhang B. L. Sun S. Xing G. M. Wang F. Li M. Y. (2016). AP2/ERF transcription factors involved in response to tomato yellow leaf curly virus in tomato. *Plant Genome* 9 1–15. 10.3835/plantgenome2015.09.0082 27898839

[B58] Hussain Z. Lata S. Kumar P. Kumar S. Tomar B. S. (2019). *Recessive Behavior of Ty-3 Gene for ToLCNDV Disease Resistance in Tomato (Solanum lycopersicum L.). Congress Communication.* Available online at: https://www.researchgate.net/profile/Kumar_Sumit3/publication/340476078_Recessive_behavior_of_Ty-3_gene_for_ToLCNDV_disease_resistance/links/5e8bf9c2a6fdcca789fbec50/Recessive-behavior-of-Ty-3-gene-for-ToLCNDV-disease-resistance.pdf (accessed May 4, 2020).

[B59] Hutton S. F. Scott J. W. Schuster D. J. (2012). Recessive resistance to tomato yellow leaf curl virus from the tomato cultivar Tyking is located in the same region as Ty-5 on chromosome 4. *Hortscience* 247 324–327. 10.21273/HORTSCI.47.3.324

[B60] Hwang I. S. Hwang B. K. (2010). The pepper 9-lipoxygenase gene CaLOX1 functions in defense and cell death responses to microbial pathogens. *Plant Physiol.* 152 948–967. 10.1104/pp.109.147827 19939946 PMC2815858

[B61] Ignacio-Espinoza J. C. Laperriere S. Yeh Y. C. Weissman J. Hou S. Long A. (2020). Ribosome-linked mRNA-rRNA chimeras reveal active novel virus-host associations. *bioRxiv* [Preprint]. 10.1101/2020.10.30.332502

[B62] Islam M. R. Hossain M. R. Jesse D. Jung H. J. Kim H. T. Park J. I. (2020). Characterization, identification and expression profiling of genome-wide R-genes in melon and their putative roles in bacterial fruit blotch resistance. *BMC Genet.* 21:80. 10.1186/s12863-020-00885-9 32698865 PMC7376666

[B63] Islam W. Noman A. Qasim M. Wang L. (2018). Plant responses to pathogen attack: small RNAs in focus. *Int. J. Mol. Sci.* 19:515. 10.3390/ijms19020515 29419801 PMC5855737

[B64] Ismayil A. Haxim Y. Wang Y. Li H. Qian L. Han T. (2018). Cotton leaf curl multan virus C4 protein suppresses both transcriptional and post-transcriptional gene silencing by interacting with SAM synthetase. *PLoS Pathog.* 14:e1007282. 10.1371/journal.ppat.1007282 30157283 PMC6133388

[B65] Jackel J. N. Storer J. M. Coursey T. Bisaro D. M. (2016). Arabidopsis RNA polymerases IV and V are required to establish H3K9 methylation, but not cytosine methylation, on geminivirus chromatin. *J. Virol.* 90 7529–7540. 10.1128/JVI.00656-16 27279611 PMC4984644

[B66] Jeevalatha A. Siddappa S. Kumar A. Kaundal P. Guleria A. Sharma S. (2017). An insight into differentially regulated genes in resistant and susceptible genotypes of potato in response to tomato leaf curl New Delhi virus-[potato] infection. *Virus Res.* 232 22–33. 10.1016/j.virusres.2017.01.015 28115198

[B67] Juárez M. Rabadán M. P. Martínez L. D. Tayahi M. Grande-Pérez A. Gómez López P. (2019). Natural hosts and genetic diversity of the emerging tomato leaf curl New Delhi virus in Spain. *Front. Microbiol.* 10:140. 10.3389/fmicb.2019.00140 30842757 PMC6391364

[B68] Juárez M. Tovar R. Fiallo-Olivé E. Aranda M. A. Gosálvez B. Castillo P. (2014). First detection of tomato leaf curl New Delhi virus infecting zucchini in Spain. *Plant Dis.* 98 857–857. 10.1094/PDIS-10-13-1050-PDN 30708660

[B69] Kachroo A. Vincelli P. Kachroo P. (2017). Signaling mechanisms underlying resistance responses: what have we learned, and how is it being applied? *Phytopathology* 107 1452–1461. 10.1094/PHYTO-04-17-0130-RVW 28609156

[B70] Kanehisa M. Goto S. (2000). KEGG: Kyoto encyclopedia of genes and genomes. *Nucleic Acids Res.* 28 27–30. 10.1093/nar/28.1.27 10592173 PMC102409

[B71] Kazan K. Manners J. M. (2011). The interplay between light and jasmonate signaling during defense and development. *J. Exp. Bot.* 62 4087–4100. 10.1093/jxb/err142 21705384

[B72] Kazlauskas D. Krupovic M. Venclovas Č (2016). The logic of DNA replication in double-stranded DNA viruses: insights from global analysis of viral genomes. *Nucleic Acids Res.* 44 4551–4564. 10.1093/nar/gkw322 27112572 PMC4889955

[B73] Koeda S. Onouchi M. Mori N. Pohan N. S. Nagano A. J. Kesumawati E. (2021). A recessive gene *pepy-1* encoding Pelota confers resistance to begomovirus isolates of PepYLCIV and PepYLCAV in *Capsicum annuum*. *Theor. Appl. Genet.* 134 2947–2964. 10.1007/s00122-021-03870-7 34081151

[B74] Kumar R. V. (2019). Plant antiviral immunity against geminiviruses and viral counter-defense for survival. *Front. Microbiol.* 10:1460. 10.3389/fmicb.2019.01460 31297106 PMC6607972

[B75] Kushwaha N. Singh A. K. Basu S. Chakraborty S. (2015). Differential response of diverse solanaceous hosts to tomato leaf curl New Delhi virus infection indicates coordinated action of NBS-LRR and RNAi-mediated host defense. *Arch. Virol.* 160 1499–1509. 10.1007/s00705-015-2399-x 25894479

[B76] Kushwaha N. K. Mansi Chakraborty S. (2017). The replication initiator protein of a geminivirus interacts with host monoubiquitination machinery and stimulates transcription of the viral genome. *PLoS Pathog.* 13:e1006587. 10.1371/journal.ppat.1006587 28859169 PMC5597257

[B77] Langfelder P. Horvath S. (2008). WGCNA: an R package for weighted correlation network analysis. *BMC Bioinformatics* 9:559. 10.1186/1471-2105-9-559 19114008 PMC2631488

[B78] Langmead B. Salzberg S. (2012). Fast gapped-read alignment with Bowtie 2. *Nat. Methods* 9 357–359. 10.1038/nmeth.1923 22388286 PMC3322381

[B79] Lapidot M. Karniel U. Gelbart D. Fogel D. Evenor D. Kutsher Y. (2015). A novel route controlling begomovirus resistance by the messenger RNA surveillance factor Pelota. *PLoS Genet.* 11:e1005538. 10.1371/journal.pgen.1005538 26448569 PMC4598160

[B80] Leibman D. Kravchik M. Wolf D. Haviv S. Weissberg M. Ophir R. (2018). Differential expression of cucumber RNA-dependent RNA polymerase 1 genes during antiviral defence and resistance. *Mol. Plant Pathol.* 19 300–312. 10.1111/mpp.12518 27879040 PMC6637986

[B81] Levy A. Tilsner J. (2020). Creating contacts between replication and movement at plasmodesmata – a role for membrane contact sites in plant virus infections? *Front. Plant Sci.* 11:862. 10.3389/fpls.2020.00862 32719692 PMC7350983

[B82] Li B. Dewey C. N. (2011). RSEM: accurate transcript quantification from RNA-Seq data with or without a reference genome. *BMC Bioinformatics* 12:323. 10.1186/1471-2105-12-323 21816040 PMC3163565

[B83] Li R. Weldegergis B. T. Li J. Jung C. Qu J. Sun Y. (2014). Virulence factors of geminivirus interact with MYC2 to subvert plant resistance and promote vector performance. *Plant Cell* 26 4991–5008. 10.1105/tpc.114.133181 25490915 PMC4311212

[B84] Li X. An M. Xia Z. Bai X. Wu Y. (2017a). Transcriptome analysis of watermelon (*Citrullus lanatus*) fruits in response to cucumber green mottle mosaic virus (CGMMV) infection. *Sci. Rep.* 7:16747. 10.1038/s41598-017-17140-4 29196660 PMC5711961

[B85] Li Y. Qin L. Zhao J. Muhammad T. Cao H. Li H. (2017b). SlMAPK3 enhances tolerance to Tomato yellow leaf curl virus (TYLCV) by regulating salicylic acid and jasmonic acid signaling in tomato (*Solanum lycopersicum*). *PLoS One* 12:e0172466. 10.1371/journal.pone.0172466 28222174 PMC5319765

[B86] Li Z. Feng Z. Maeli M. Jian Y. Sheng Y. H. (2017c). Jasmonate signaling and manipulation by pathogens and insects. *J. Exp. Bot.* 68 1371–1385. 28069779 10.1093/jxb/erw478 PMC6075518

[B87] Liu Q. Liu H. Gong Y. Tao Y. Jiang L. Zuo W. (2017). An atypical thioredoxin imparts early resistance to sugarcane mosaic virus in maize. *Mol. Plant.* 10 483–497. 10.1016/j.molp.2017.02.002 28216424

[B88] López C. Ferriol M. Picó M. B. (2015). Mechanical transmission of tomato leaf curl New Delhi virus to cucurbit germplasm: selection of tolerance sources in *Cucumis melo*. *Euphytica* 204 679–691. 10.1007/s10681-015-1371-x

[B89] Lou L. Su X. Liu X. Liu Z. (2020). Transcriptome analysis of *Luffa cylindrica* (L.) Roem response to infection with cucumber mosaic virus (CMV). *Gene* 737:144451. 10.1016/j.gene.2020.144451 32035243

[B90] Love M. I. Huber W. Anders S. (2014). Moderated estimation of fold change and dispersion for RNA-seq data with DESeq2. *Genome Biol.* 15:550. 10.1186/s13059-014-0550-8 25516281 PMC4302049

[B91] Lozano-Durán R. Rosas-Díaz T. Gusmaroli G. Luna A. P. Taconnat L. Deng X. W. (2011). Geminiviruses subvert ubiquitination by altering CSN-mediated derubylation of SCF E3 ligase complexes and inhibit jasmonate signaling in *Arabidopsis thaliana*. *Plant Cell* 23 1014–1032. 10.1105/tpc.110.080267 21441437 PMC3082251

[B92] Lu S. Faris J. D. Edwards M. C. (2017). Molecular cloning and characterization of two novel genes from hexaploid wheat that encode double PR-1 domains coupled with a receptor-like protein kinase. *Mol. Genet. Genomics* 292 435–452. 10.1007/s00438-017-1287-3 28120099

[B93] Luna-Rivero M. S. Hernández-Zepeda C. Villanueva-Alonzo H. Minero-García Y. Castell-González S. E. Moreno-Valenzuela O. A. (2016). Expression of genes involved in the salicylic acid pathway in type h1 thioredoxin transiently silenced pepper plants during a begomovirus compatible interaction. *Mol. Genet. Genomics* 291 819–830. 10.1007/s00438-015-1148-x 26606929

[B94] Machado J. P. B. Calil I. P. Santos A. A. Fontes E. P. B. (2017). Translational control in plant antiviral immunity. *Genet. Mol. Biol.* 40 292–304. 10.1590/1678-4685-GMB-2016-0092 28199446 PMC5452134

[B95] Maio F. Helderman T. A. Arroyo-Mateos M. van der Wolf M. Boeren S. Prins M. (2020). Identification of tomato proteins that interact with Replication initiator protein (Rep) of the geminivirus TYLCV. *Front. Plant Sci.* 11:1069. 10.3389/fpls.2020.01069 32760417 PMC7373745

[B96] Mäkinen K. De S. (2019). The significance of methionine cycle enzymes in plant virus infections. *Curr. Opin. Plant Biol.* 50 67–75. 10.1016/j.pbi.2019.03.002 30959442

[B97] Mallory A. Vaucheret H. (2010). Form, function, and regulation of ARGONAUTE proteins. *Plant Cell* 22 3879–3889. 10.1105/tpc.110.080671 21183704 PMC3027166

[B98] Martín-Hernández A. M. Picó B. (2021). Natural resistances to viruses in cucurbits. *Agronomy* 11:23. 10.3390/agronomy11010023

[B99] Mathioudakis M. M. Khechmar S. Owen C. A. Medina V. Ben Mansour K. Tomaszewska W. (2018). A thioredoxin domain-containing protein interacts with pepino mosaic virus triple gene block protein 1. *Int. J. Mol. Sci.* 19:3747. 10.3390/ijms19123747 30477269 PMC6320799

[B100] McCreight J. D. Liu H.-Y. Turini T. A. (2008). Genetic resistance to cucurbit leaf crumple virus in melon. *Hortscience* 43 122–126. 10.21273/HORTSCI.43.1.122

[B101] Messelink G. J. Calvo F. J. Marín F. Janssen D. (2020). “Cucurbits,” in *Integrated Pest and Disease Management in Greenhouse Crops*, eds Gullino M. L. Albajes R. Nicot P. C. (Cham: Springer International Publishing), 537–566. 10.1007/978-3-030-22304-5_19

[B102] Mochizuki T. Ogata Y. Hirata Y. Ohki S. T. (2014). Quantitative transcriptional changes associated with chlorosis severity in mosaic leaves of tobacco plants infected with cucumber mosaic virus. *Mol. Plant Pathol.* 15 242–254. 10.1111/mpp.12081 24745045 PMC6638806

[B103] Morata J. Puigdomènech P. (2017). Variability among *Cucurbitaceae* species (melon, cucumber and watermelon) in a genomic region containing a cluster of NBS-LRR genes. *BMC Genomics* 18:138. 10.1186/s12864-017-3529-5 28178932 PMC5299730

[B104] Moriones E. Praveen S. Chakraborty S. (2017). Tomato leaf curl New Delhi virus: an emerging virus complex threatening vegetable and fiber crops. *Viruses* 9:264. 10.3390/v9100264 28934148 PMC5691616

[B105] Musidlak O. Nawrot R. Goździcka-Józefiak A. (2017). Which plant proteins are involved in antiviral defense? Review on in vivo and in vitro activities of selected plant proteins against viruses. *Int. J. Mol. Sci.* 18:2300. 10.3390/ijms18112300 29104238 PMC5713270

[B106] Naqvi A. R. Haq Q. M. Mukherjee S. K. (2010). MicroRNA profiling of tomato leaf curl New Delhi virus (tolcndv) infected tomato leaves indicates that deregulation of mir159/319 and mir172 might be linked with leaf curl disease. *Virol. J.* 7:281. 10.1186/1743-422X-7-281 20973960 PMC2972279

[B107] Naqvi R. Z. Zaidi S. S. E. A. Akhtar K. P. Strickler S. Woldemariam M. Mishra B. (2017). Transcriptomics reveals multiple resistance mechanisms against cotton leaf curl disease in a naturally immune cotton species, *Gossypium arboreum*. *Sci. Rep.* 7:15880. 10.1038/s41598-017-15963-9 29162860 PMC5698292

[B108] Nemchinov L. Boutanaev A. Postnikova O. (2016). Virus-induced gene silencing of the RPC5-like subunit of RNA polymerase III caused pleiotropic effects in *Nicotiana benthamiana*. *Sci. Rep.* 6:27785. 10.1038/srep27785 27282827 PMC4901293

[B109] Nueda M. J. Ferrer A. Conesa A. (2012). ARSyN: a method for the identification and removal of systematic noise in multifactorial time course microarray experiments. *Biostatistics* 13 553–566. 10.1093/biostatistics/kxr042 22085896

[B110] Pandey S. Muthamilarasan M. Sharma N. Chaudhry V. Dulani P. Shweta S. (2019). Characterization of DEAD-box family of RNA helicases in tomato provides insights into their roles in biotic and abiotic stresses. *Environ. Exp. Bot.* 158 107–116. 10.1016/j.envexpbot.2018.11.018

[B111] Panno S. Caruso A. G. Troiano E. Luigi M. Manglli A. Vatrano T. (2019). Emergence of tomato leaf curl New Delhi virus in Italy: estimation of incidence and genetic diversity. *Plant Pathol.* 68 601–608. 10.1111/ppa.12978

[B112] Panno S. Iacono G. Davino M. Marchione S. Zappardo V. Bella P. (2016). First report of tomato leaf curl New Delhi virus affecting zucchini squash in an important horticultural area of southern Italy. *New Dis. Rep.* 33:6. 10.5197/j.2044-0588.2016.033.006

[B113] Pérez-de-Castro A. López-Martín M. Esteras C. Garcés-Claver A. Palomares-Ríus F. J. Picó M. B. (2020). Melon genome regions associated with TGR-1551-derived resistance to cucurbit yellow stunting disorder virus. *Int. J. Mol. Sci.* 21:5970. 10.3390/ijms21175970 32825131 PMC7504372

[B114] Pitrat M. (2016). “Melon genetic resources: phenotypic diversity and horticultural taxonomy,” in *Genetics and Genomics of Cucurbitaceae*, Vol. 20 eds Grumet R. Katzir N. Garcia-Mas J. (Cham: Springer Nature), 25–59. 10.1007/7397_2016_10

[B115] Pradhan B. Van Tien V. Dey N. Mukherjee S. K. (2017). *Molecular Biology of Geminivirus DNA Replication. Viral Replication, 2-35 Avid Science.* Available online at: https://www.researchgate.net/profile/Tien-Vu-6/publication/316456253_Molecular_Biology_of_Geminivirus_DNA_Replication/links/5906ecf0aca272116d333677/Molecular-Biology-of-Geminivirus-DNA-Replication.pdf (accessed December, 2021).

[B116] Prakash V. Singh A. Singh A. K. Dalmay T. Chakraborty S. (2020). Tobacco RNA-dependent RNA polymerase 1 affects the expression of defence-related genes in *Nicotiana benthamiana* upon tomato leaf curl Gujarat virus infection. *Planta* 252:11. 10.1007/s00425-020-03417-y 32613448

[B117] Preiss W. Jeske H. (2003). Multitasking in replication is common among geminiviruses. *J. Virol.* 77 2972–2980. 10.1128/jvi.77.5.2972-2980.2003 12584322 PMC149778

[B118] Prins M. W. Van Enckevort L. J. G. Versluis H. P. (2019). *Geminivirus Resistant Plants*. *U.S. Patent No. 2020/0392530 A1.* Wageningen, NL: International application published under the patent cooperation treaty (PCT).

[B119] Quezada E. H. García G. X. Arthikala M. K. Melappa G. Lara M. Nanjareddy K. (2019). Cysteine-rich receptor-like kinase gene family identification in the *Phaseolus* genome and comparative analysis of their expression profiles specific to mycorrhizal and rhizobial symbiosis. *Genes* 10:59. 10.3390/genes10010059 30658517 PMC6356535

[B120] Qui X. Bao F. S. Xie Z. (2009). Small RNA deep sequencing reveals role for *Arabidopsis thaliana* RNA-dependent RNA polymerases in viral siRNA biogenesis. *PLoS One* 4:e4971. 10.1371/journal.pone.0004971 19308254 PMC2654919

[B121] Rai N. Sahu P. Gupta S. Reddy M. K. Ravishankar K. Singh M. (2013). Identification and validation of an ISSR marker linked to tomato leaf curl New Delhi virus resistant gene in a core set of tomato accessions. *Vegetable Sci.* 40 1–6.

[B122] Raja P. Jackel J. N. Li S. Heard I. M. Bisaro D. M. (2014). Arabidopsis double-stranded RNA binding protein DRB3 participates in methylation-mediated defense against geminiviruses. *J. Virol.* 88 2611–2622. 10.1128/JVI.02305-13 24352449 PMC3958096

[B123] Ramesh S. V. Sahu P. P. Prasad M. Praveen S. Pappu H. R. (2017). Geminiviruses and plant hosts: a closer examination of the molecular arms race. *Viruses* 9:256. 10.3390/v9090256 28914771 PMC5618022

[B124] Ranji A. Boris-Lawrie K. (2010). RNA helicases: emerging roles in viral replication and the host innate response. *RNA Biol.* 7 775–787. 10.4161/rna.7.6.14249 21173576 PMC3073335

[B125] Rizvi I. Choudhury N. R. Tuteja N. (2015). Insights into the functional characteristics of geminivirus rolling-circle replication initiator protein and its interaction with host factors affecting viral DNA replication. *Arch. Virol.* 160 375–387. 10.1007/s00705-014-2297-7 25449306

[B126] Rodríguez-Negrete E. Lozano-Durán R. Piedra-Aguilera A. Cruzado L. Bejarano E. R. Castillo A. G. (2013). Geminivirus Rep protein interferes with the plant DNA methylation machinery and suppresses transcriptional gene silencing. *New Phytol.* 199 464–475. 10.1111/nph.12286 23614786

[B127] Román B. Gómez P. Picó B. López C. Janssen D. (2019). Candidate gene analysis of tomato leaf curl New Delhi virus resistance in *Cucumis melo*. *Sci. Hortic.* 243 12–20. 10.1016/j.scienta.2018.07.005

[B128] Romay G. Pitrat M. Lecoq H. Wipf-Scheibel C. Millot P. Girardot G. (2019). Resistance against melon chlorotic mosaic virus and tomato leaf curl New Delhi virus in melon. *Plant Dis.* 103 2913–2919. 10.1094/PDIS-02-19-0298-RE 31436474

[B129] Rossitto de Marchi B. Kinene T. Krause-Sakate R. Boykin L. M. Ndunguru J. Kehoe M. (2020). Genetic diversity and SNP’s from the chloroplast coding regions of virus-infected cassava. *PeerJ* 8:e8632. 10.7717/peerj.8632 32175188 PMC7058106

[B130] Roy A. Bal S. S. Fergany M. Kaur S. Singh H. Malik A. A. (2012). Wild melon diversity in India (Punjab state). *Genet. Resour. Crop Evol.* 59 755–767. 10.1007/s10722-011-9716-3

[B131] Sade D. Sade N. Brotman Y. Czosnek H. (2020). Tomato yellow leaf curl virus (TYLCV)-resistant tomatoes share molecular mechanisms sustaining resistance with their wild progenitor *Solanum habrochaites* but not with TYLCV-susceptible tomatoes. *Plant Sci. Int. J. Exp. Plant Biol.* 295:110439. 10.1016/j.plantsci.2020.110439 32534617

[B132] Sáez C. Ambrosio L. G. M. Miguel S. M. Valcárcel J. V. Díez M. J. Picó B. (2021). Resistant sources and genetic control of resistance to ToLCNDV in cucumber. *Microorganisms* 9:913. 10.3390/microorganisms9050913 33923281 PMC8146778

[B133] Sáez C. Esteras C. Martínez C. Ferriol M. Dhillon N. P. López C. (2017). Resistance to tomato leaf curl New Delhi virus in melon is controlled by a major QTL located in chromosome 11. *Plant Cell Rep.* 36 1571–1584. 10.1007/s00299-017-2175-3 28710536

[B134] Sáez C. Martínez C. Ferriol M. Manzano S. Velasco L. Jamilena M. (2016). Resistance to tomato leaf curl New Delhi virus in *Cucurbita* spp. *Ann. Appl. Biol.* 169 91–105.

[B135] Sáez C. Martínez C. Montero-Pau J. Esteras C. Sifres A. Blanca J. (2020). A major QTL located in chromosome 8 of *Cucurbita moschata* is responsible for resistance to tomato leaf curl New Delhi virus. *Front. Plant Sci.* 11:207. 10.3389/fpls.2020.00207 32265946 PMC7100279

[B136] Sahu P. P. Rai N. K. Chakraborty S. Singh M. Chandrappa P. H. Ramesh B. (2010). Tomato cultivar tolerant to tomato leaf curl New Delhi virus infection induces virus-specific short interfering RNA accumulation and defence-associated host gene expression. *Mol. Plant Pathol.* 11 531–544. 10.1111/j.1364-3703.2010.00630.x 20618710 PMC6640424

[B137] Sahu P. P. Sharma N. Puranik S. Chakraborty S. Prasad M. (2016). Tomato 26S proteasome subunit RPT4a regulates ToLCNDV transcription and activates hypersensitive response in tomato. *Sci. Rep.* 6:27078. 10.1038/srep27078 27252084 PMC4890432

[B138] Sanfaçon H. (2015). Plant translation factors and virus resistance. *Viruses* 7 3392–3419. 10.3390/v7072778 26114476 PMC4517107

[B139] Saunders K. Lucy A. Stanley J. (1992). RNA-primed complementary-sense DNA synthesis of the geminivirus African cassava mosaic virus. *Nucleic Acids Res.* 20 6311–6315. 10.1093/nar/20.23.6311 1475192 PMC334521

[B140] Schmittgen T. D. Livak K. J. (2008). Analyzing real-time PCR data by the comparative CT method. *Nat. Protoc.* 3 1101–1108. 10.1038/nprot.2008.73 18546601

[B141] Schommer C. Palatnik J. F. Aggarwal P. Chételat A. Cubas P. Farmer E. E. (2008). Control of jasmonate biosynthesis and senescence by miR319 targets. *PLoS Biol.* 6:e230. 10.1371/journal.pbio.0060230 18816164 PMC2553836

[B142] Seo H. S. Song J. T. Cheong J. J. Lee Y. H. Lee Y. W. Hwang I. (2001). Jasmonic acid carboxyl methyltransferase: a key enzyme for jasmonate-regulated plant responses. *Proc. Natl. Acad. Sci. U.S.A.* 98 4788–4793. 10.1073/pnas.081557298 11287667 PMC31912

[B143] Shang J. Xi D. H. Xu F. Wang S. D. Cao S. Xu M. Y. (2011). A broad-spectrum, efficient and nontransgenic approach to control plant viruses by application of salicylic acid and jasmonic acid. *Planta* 233 299–308. 10.1007/s00425-010-1308-5 21046144

[B144] Sharma A. Boris-Lawrie K. (2012). Determination of host RNA helicases activity in viral replication. *Methods Enzymol.* 511 405–435. 10.1016/B978-0-12-396546-2.00019-X 22713331 PMC4862593

[B145] Sharma N. Sahu P. P. Prasad A. Muthamilarasan M. Waseem M. Khan Y. (2021). The *Sw5a* gene confers resistance to ToLCNDV and triggers an HR response after direct AC4 effector recognition. *Proc. Natl. Acad. Sci. U.S.A.* 118:e2101833118. 10.1073/pnas.2101833118 34385303 PMC8379908

[B146] Souza P. García-Ruíz H. Carvalho F. (2019). What proteomics can reveal about plant-virus interactions? Photosynthesis-related proteins on the spotlight. *Theor. Exp. Plant Physiol.* 31 227–248. 10.1007/s40626-019-00142-0 31355128 PMC6660014

[B147] Sun X. Wang Z. Gu Q. Li H. Han W. Shi Y. (2017). Transcriptome analysis of *Cucumis sativus* infected by cucurbit chlorotic yellows virus. *Virol. J.* 14:18. 10.1186/s12985-017-0690-z 28148297 PMC5288851

[B148] Sun Y. Fan M. He Y. (2019). Transcriptome analysis of watermelon leaves reveals candidate genes responsive to cucumber green mottle mosaic virus infection. *Int. J. Mol. Sci.* 20:610. 10.3390/ijms20030610 30708960 PMC6387395

[B149] Supek F. Bošnjak M. Škunca N. Šmuc T. (2011). REVIGO summarizes and visualizes long lists of gene ontology terms. *PLoS One* 6:e21800. 10.1371/journal.pone.0021800 21789182 PMC3138752

[B150] Suyal G. Mukherjee S. K. Srivastava P. S. Choudhury N. R. (2013). *Arabidopsis thaliana* MCM2 plays role(s) in mungbean yellow mosaic India virus (MYMIV) DNA replication. *Arch. Virol.* 158 981–992. 10.1007/s00705-012-1563-9 23242774

[B151] Tsai W. A. Weng S. H. Chen M. C. Lin J. S. Tsai W. S. (2019). Priming of plant resistance to heat stress and tomato yellow leaf curl Thailand virus with plant-derived materials. *Front. Plant Sci.* 10:906. 10.3389/fpls.2019.00906 31354773 PMC6640737

[B152] Ullah R. Akhtar K. P. Moffett P. Mansoor S. Briddon R. W. Saeed M. (2014). An analysis of the resistance of *Gossypium arboreum* to cotton leaf curl disease by grafting. *Eur. J. Plant Pathol.* 139 837–847. 10.1007/s10658-014-0437-2

[B153] Untergasser A. Nijveen H. Rao X. Bisseling T. Geurts R. Jack A. M. (2007). Leunissen: Primer3Plus, an enhanced web interface to Primer3. *Nucleic Acids Res.* 35 W71–W74. 10.1093/nar/gkm306 17485472 PMC1933133

[B154] Verlaan M. G. Hutton S. F. Ibrahem R. M. Kormelink R. Visser R. G. Scott J. W. (2013). The tomato yellow leaf curl virus resistance genes *Ty-1* and *Ty-3* are allelic and code for DFDGD-class RNA–dependent RNA polymerases. *PLoS Genet.* 9:e1003399. 10.1371/journal.pgen.1003399 23555305 PMC3610679

[B155] Vicente J. Cascon T. Vicedo B. Garcia-Agustin P. Hamberg M. Castresana C. (2012). Role of 9-lipoxygenase and alpha-dioxygenase oxylipin pathways as modulators of local and systemic defense. *Mol. Plant* 5 914–928. 10.1093/mp/ssr105 22199234

[B156] Vinutha T. Kumar G. Garg V. Canto T. Palukaitis P. Ramesh S. V. (2018). Tomato geminivirus encoded RNAi suppressor protein, AC4 interacts with host AGO4 and precludes viral DNA methylation. *Gene* 678 184–195. 10.1016/j.gene.2018.08.009 30081188

[B157] Vinutha T. Vanchinathan S. Bansal N. Kumar G. Permar V. Watts A. (2020). Tomato auxin biosynthesis/signaling is reprogrammed by the geminivirus to enhance its pathogenicity. *Planta* 252:51. 10.1007/s00425-020-03452-9 32940767

[B158] Wasternack C. Song S. (2017). Jasmonates: biosynthesis, metabolism, and signaling by proteins activating and repressing transcription. *J. Exp. Bot.* 68 1303–1321. 10.1093/jxb/erw443 27940470

[B159] Willmann M. R. Endres M. W. Cook R. T. Gregory B. D. (2011). The functions of RNA-dependent RNA polymerases in *Arabidopsis*. *Arabidopsis Book* 9:e0146. 10.1199/tab.0146 22303271 PMC3268507

[B160] Wu M. Ding X. Fu X. Lozano-Durán R. (2019). Transcriptional reprogramming caused by the geminivirus tomato yellow leaf curl virus in local or systemic infections in *Nicotiana benthamiana*. *BMC Genomics* 20:542. 10.1186/s12864-019-5842-7 31272383 PMC6611054

[B161] Xia C. Li S. Hou W. Fan Z. Xiao H. Lu M. (2017). Global transcriptomic changes induced by infection of cucumber (*Cucumis sativus* L.) with mild and severe variants of Hop stunt viroid. *Front. Microbiol.* 8:2427. 10.3389/fmicb.2017.02427 29312160 PMC5733102

[B162] Xiong W. Lan T. Mo B. (2021). Extraribosomal functions of cytosolic ribosomal proteins in plants. *Front. Plant Sci.* 12:607157. 10.3389/fpls.2021.607157 33968093 PMC8096920

[B163] Yadeta K. A. Elmore J. M. Creer A. Y. Feng B. Franco J. Y. Rufian J. S. (2017). A cysteine-rich protein kinase associates with a membrane immune complex and the cysteine residues are required for cell death. *Plant Physiol.* 173 771–787. 10.1104/pp.16.01404 27852951 PMC5210739

[B164] Yan C. Xie D. (2015). Jasmonate in plant defense: sentinel or double agent? *Plant Biotechnol. J.* 13 1233–1240. 10.1111/pbi.12417 26096226

[B165] Yan Z. Wolters A. M. A. Navas-Castillo J. Bai Y. (2021). The global dimension of tomato yellow leaf curl disease: current status and breeding perspectives. *Microorganisms* 9:740. 10.3390/microorganisms9040740 PMC8066563 33916319

[B166] Yang S. J. Carter S. A. Cole A. B. Cheng N. H. Nelson R. S. (2004). A natural variant of a host RNA-dependent RNA polymerase is associated with increased susceptibility to viruses by *Nicotiana benthamiana*. *Proc. Natl. Acad. Sci. U.S.A.* 101 6297–6302. 10.1073/pnas.0304346101 15079073 PMC395963

[B167] You W. J. Feng Y. R. Shen Y. H. Chen Y. R. Chen T. Y. Fu S. F. (2019). Silencing of NbCMT3s has pleiotropic effects on development by interfering with autophagy-related genes in *Nicotiana benthamiana*. *Plant Cell Physiol.* 60 1120–1135. 10.1093/pcp/pcz034 30785195

[B168] Yousif M. T. Kheyr-Pour A. Gronenborn B. Pitrat M. Dogimont C. (2007). Sources of 536 resistance to watermelon chlorotic stunt virus in melon. *Plant Breed.* 126 422–427. 10.1111/j.1439-0523.2007.01366.x

[B169] Yu G. Wang L. Han Y. He Q. (2012). ClusterProfiler: an R package for comparing biological themes among gene clusters. *OMICS* 16 284–287. 10.1089/omi.2011.0118 22455463 PMC3339379

[B170] Zaidi S. S. Naqvi R. Z. Asif M. Strickler S. Shakir S. Shafiq M. (2020). Molecular insight into cotton leaf curl geminivirus disease resistance in cultivated cotton (*Gossypium hirsutum*). *Plant Biotechnol. J.* 18 691–706. 10.1111/pbi.13236 31448544 PMC7004920

[B171] Zhai Y. Peng H. Neff M. M. Pappu H. R. (2020). Emerging molecular links between plant photomorphogenesis and virus resistance. *Front. Plant Sci.* 11:920. 10.3389/fpls.2020.00920 32695129 PMC7338571

[B172] Zhan J. Shi H. Li W. Zhang C. Zhang Y. (2021). NbTMP14 is involved in tomato spotted wilt virus infection and symptom development by interaction with the viral NSm protein. *Viruses* 13:427. 10.3390/v13030427 33800072 PMC7999277

[B173] Zhang L. Zhang F. Melotto M. Yao J. He S. Y. (2017). Jasmonate signaling and manipulation by pathogens and insects. *J. Exp. Bot.* 68 1371–1385. 10.1093/jxb/erw478 28069779 PMC6075518

[B174] Zhang Z. Chen H. Huang X. Xia R. Zhao Q. Lai J. (2011). BSCTV C2 attenuates the degradation of SAMDC1 to suppress DNA methylation-mediated gene silencing in *Arabidopsis*. *Plant Cell* 23 273–288. 10.1105/tpc.110.081695 21245466 PMC3051253

[B175] Zorzatto C. Machado J. P. B. Lopes K. V. Nascimento K. J. Pereira W. A. Brustolini O. J. (2015). NIK1-mediated translation suppression functions as a plant antiviral immunity mechanism. *Nature* 520 679–682. 10.1038/nature14171 25707794 PMC4779052

