# Supplementary figures and images for: RNA-Seq Transcriptome Analysis Provides Candidate Genes for Resistance to Tomato Leaf Curl New Delhi Virus in Melon
Source: Front Plant Sci. 2022 Jan 18;12:798858. doi: 10.3389/fpls.2021.798858 (PMC8805612; doi:10.3389/fpls.2021.798858)

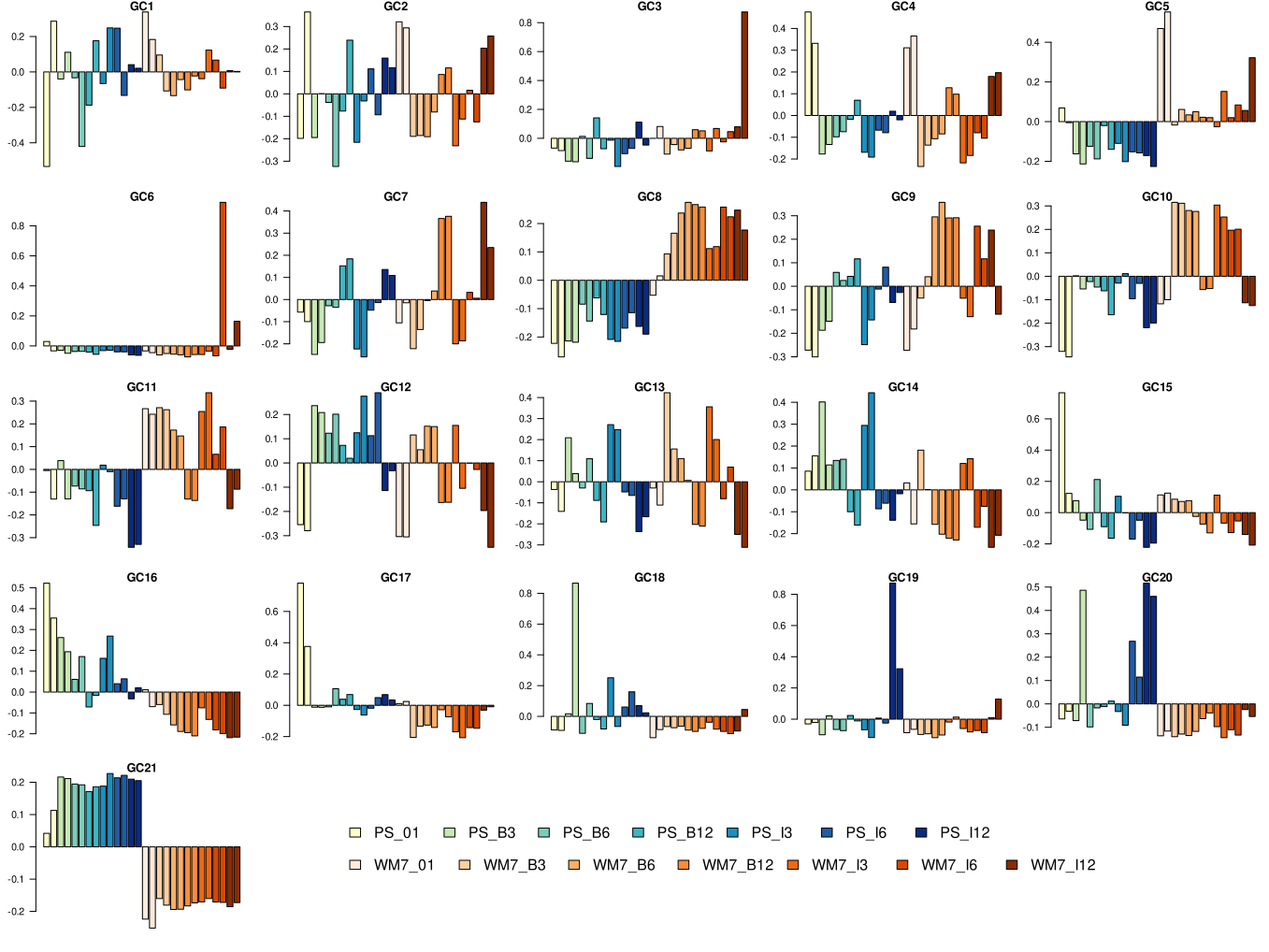

Supplement: Supplementary Figure 1 — Gene expression patterns of the analyzed samples for each gene cluster. Eigengene values are represented across tissues. [file Image_1.PNG]
